# A novel LncRNA PTH-AS upregulates interferon-related DNA damage resistance signature genes and promotes metastasis in human breast cancer xenografts

**DOI:** 10.1016/j.jbc.2022.102065

**Published:** 2022-05-23

**Authors:** Miho Akimoto, Takao Susa, Noriyuki Okudaira, Harumi Hisaki, Masayoshi Iizuka, Hiroko Okinaga, Tomoki Okazaki, Mimi Tamamori-Adachi

**Affiliations:** 1 Department of Biochemistry, Teikyo University School of Medicine, Tokyo, Japan; 2 General Medical Education and Research Center, Teikyo University, Tokyo, Japan; 3 Medical Education Center, Teikyo University School of Medicine, Tokyo, Japan; 4 Department of Internal Medicine, Teikyo University School of Medicine, Tokyo, Japan

**Keywords:** long noncoding RNA, signal transducer and activator of transcription 1, breast cancer, metastasis, parathyroid hormone, interferon-related DNA damage resistance signature, BSA, bovine serum albumin, cDNA, complementary DNA, DMEM, Dulbecco’s modified Eagle's medium, DXR, doxorubicin, ECM, extracellular matrix, EMT, epithelial–mesenchymal transition, GO, Gene Ontology, IRDS, interferon-related DNA damage resistance signature, ISG, interferon-stimulated gene, JAK, Janus kinase, lncRNA, long noncoding RNA, MFH, malignant fibrous histiocytoma, PA, parathyroid adenoma, pSTAT1, phosphorylated STAT1, PTH, parathyroid hormone, qRT–PCR, quantitative RT–PCR, RACE, rapid amplification of complementary DNA ends, STAT1, signal transducer and activator of transcription 1, TAM, tumor-associated macrophage, tSTAT1, total STAT1, VEGF, vascular endothelial growth factor

## Abstract

Long noncoding RNAs (lncRNAs) are important tissue-specific regulators of gene expression, and their dysregulation can induce aberrant gene expression leading to various pathological conditions, including cancer. Although many lncRNAs have been discovered by computational analysis, most of these are as yet unannotated. Herein, we describe the nature and function of a novel lncRNA detected downstream of the human parathyroid hormone (PTH) gene in both extremely rare ectopic PTH-producing retroperitoneal malignant fibrous histiocytoma and parathyroid tumors with PTH overproduction. This novel lncRNA, which we have named “PTH-AS,” has never been registered in a public database, and here, we investigated for the first time its exact locus, length, transcription direction, polyadenylation, and nuclear localization. Microarray and Gene Ontology analyses demonstrated that forced expression of PTH-AS in PTH–nonexpressing human breast cancer T47D cells did not induce the ectopic expression of the nearby PTH gene but did significantly upregulate Janus kinase–signal transducer and activator of transcription pathway–related genes such as cancer-promoting interferon-related DNA damage resistance signature (IRDS) genes. Importantly, we show that PTH-AS expression not only enhanced T47D cell invasion and resistance to the DNA-damaging drug doxorubicin but also promoted lung metastasis rather than tumor growth in a mouse xenograft model. In addition, PTH-AS–expressing T47D tumors showed increased macrophage infiltration that promoted angiogenesis, similar to IRDS-associated cancer characteristics. Although the detailed molecular mechanism remains imperfectly understood, we conclude that PTH-AS may contribute to tumor development, possibly through IRDS gene upregulation.

Noncoding RNAs longer than 200 nucleotides are classified as long coding RNAs (lncRNAs), and the current release of the public database LNCipedia contains about 128,000 transcripts and about 57,000 genes (1). Recent studies have shown that lncRNAs regulate tissue-specific gene expression through interaction with DNA, proteins, and RNA, thereby driving vital processes, such as differentiation, development, and immunoregulation (2, 3, 4, 5). Usually, lncRNA expression is tightly controlled and fine tuned, but dysregulation causes a variety of diseases, including cancer. In fact, next-generation sequencing transcriptomes have revealed that thousands of lncRNAs are abnormally expressed in various cancers (6, 7, 8, 9, 10). Some lncRNAs affect cancer phenotypes and promote ultimately catastrophic metastasis (7, 8, 9, 10). While lncRNAs are receiving increased attention, most are predicted only by computational analysis and very few are accurately annotated (11). Therefore, there is great interest not only in revealing the existence of predictive lncRNAs but also in further explaining their effects on cellular processes and phenotypes.

We previously reported the possible existence of a new lncRNA located downstream of the human parathyroid hormone (PTH) gene in ectopic PTH-producing malignant fibrous histiocytoma (MFH) (12). Interestingly, the same lncRNA was also detected in a PTH-overexpressing parathyroid adenoma (PA) from another patient, suggesting that its expression is closely related to the expression of the nearby PTH gene. However, the exact location and function of the lncRNA was not known. Herein, we describe the characteristics of this lncRNA and the effect of forced expression on PTH–nonexpressing human breast cancer T47D cells on global gene expression including the PTH gene by microarray analysis. Moreover, based on Gene Ontology (GO) analysis, the effects of expression of the lncRNA on cell function, especially cancer phenotype, are demonstrated *in vitro* and *in vivo*. In this study, the lncRNA of interest was identified as an intergenic lncRNA, which we named “PTH-AS” because it is transcribed from the antisense strand downstream of the PTH gene. PTH-AS expression in T47D cells unexpectedly did not induce PTH expression but instead dramatically upregulated signal transducer and activator of transcription 1 (STAT1) and its downstream genes, especially interferon-related DNA damage resistance signature (IRDS).

The IRDS genes belong to a subset of interferon-stimulated genes (ISGs) and include *IFI44*, *OAS1*, *OAS2*, *ISG15*, *MX1*, *IFIT1*, and *IFIT3*. Unlike other Janus kinase (JAK)–STAT signaling–regulated genes, IRDS expression is induced *via* the unphosphorylated STAT1/STAT2/IRF9 complex (U-ISGF3) rather than the phosphorylated complex (ISGF3) (13, 14). IRDS is strongly associated with resistance to radiation therapy and antitumor treatment with DNA-damaging agents, including doxorubicin (DXR) and cisplatin, because it induces resistance to DNA damage. Indeed, the expression of IRDS in tumor tissue correlates with poor prognosis and has recently attracted attention as a marker for the diagnosis and prognosis of various cancers, including breast cancer (15, 16). In addition, although the detailed mechanism has not been elucidated, IRDS in the tumor microenvironment acts on both tumor cells and stromal cells, promoting epithelial–mesenchymal transition (EMT) and reducing T-cell cytotoxicity. Therefore, IRDS increases tumor aggressiveness in addition to anticancer drug resistance.

The results of this study revealed that PTH-AS expression in T47D cells enhanced EMT-like alterations, invasiveness, and resistance to DXR, similar to the situation reported for IRDS. In a mouse xenograft model, PTH-AS expression promoted lung metastases rather than T47D tumor growth and simultaneously increased macrophage infiltration and associated angiogenesis. These results demonstrate that PTH-AS is involved in malignant transformation and anticancer drug resistance.

## Results

### Identification of a novel lncRNA downstream of the PTH gene

**Figure 1:**
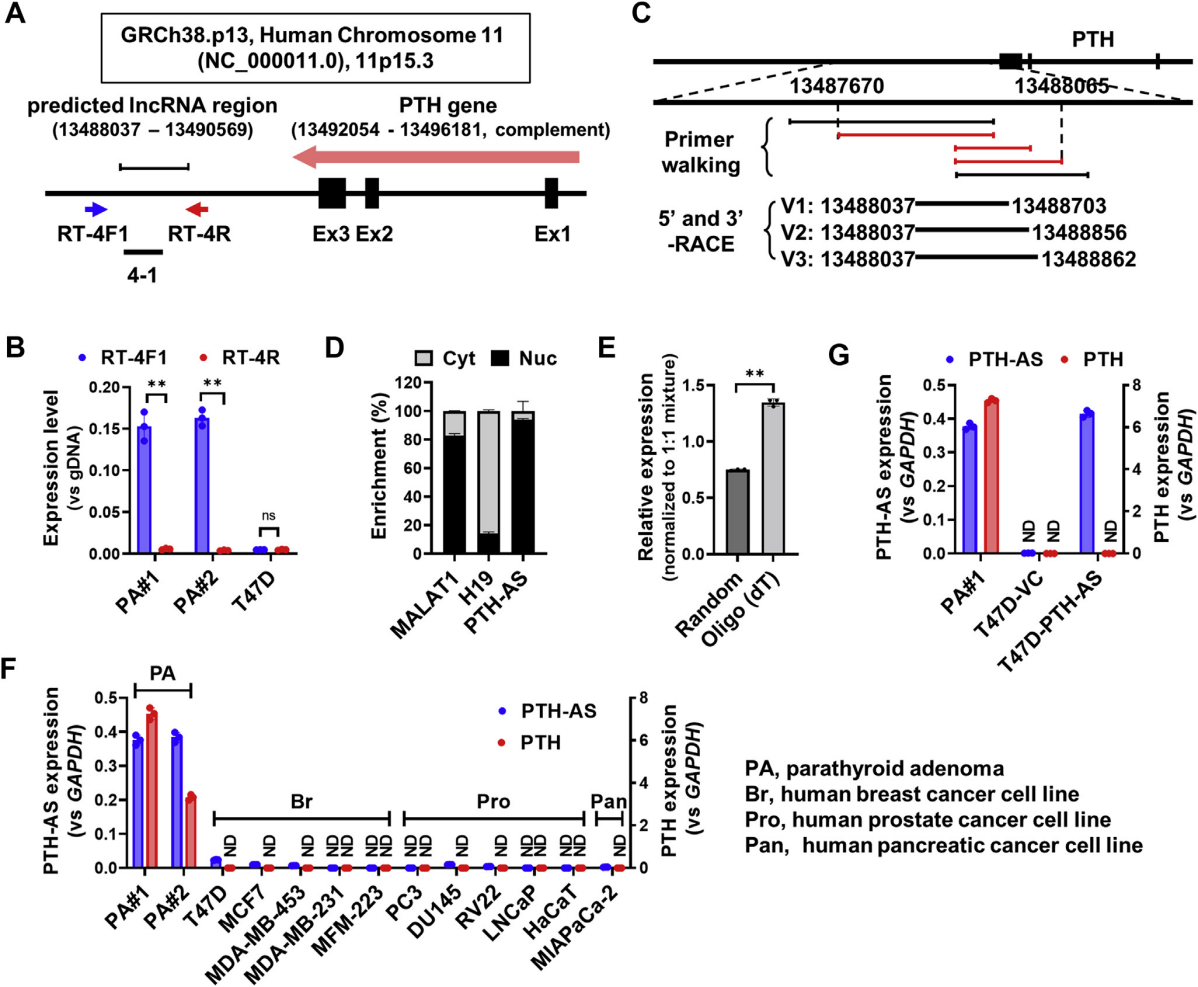
**Determining the nature of novel lncRNAs downstream of the PTH gene.***A*, predicted position of the target lncRNA based on the sequence information of GR Ch38.p13, human chromosome 11 (NC_000011.10). *B*, determination of the transcription direction of lncRNA. Total RNA from parathyroid adenomas (PA #1 and PA #2) expressing the lncRNA was used as a template for reverse transcription (RT). After strand-specific RT using the sense primer RT-4F1 or antisense primer RT-4R shown in (*A*), qPCR was performed using the primer set for the 4-1 fragment. The data are shown as relative values, and the 4-1 fragment amplification level when the genomic DNA (gDNA) of each sample is used as a template is set to 1 (n = 3). Human breast cancer T47D cells nonexpressing the lncRNA were used as a negative control. *C*, identification of the target lncRNA position by primer walking and determination of transcript position by 5ʹ- and 3ʹ-RACE. Detailed information on the primers used for strand-specific RT and primer walking is shown in Table S1. PCR fragments amplified by primer walking are indicated by a *red line*, and those that are not amplified are indicated by a *black line*. V1, V2, and V3 represent the three variants of the lncRNA identified by RACE. *D,* intracellular distribution of PTH-AS. The amount of PTH-AS in the nuclear or cytoplasmic fraction was quantified by qRT–PCR with a 4-1 primer pair and is shown as a relative ratio (n = 3). MALAT1 and H19 were used as positive controls for nuclear and cytoplasmic lncRNAs, respectively. *E*, using the RNA of PA expressing PTH-AS as a template, RT was performed in the presence of random hexamer (poly(A) tail independent) or oligo(dT)_18_ (poly(A) tail dependent). The resulting cDNA was used to determine the amplification level of PTH-AS by qRT–PCR and normalized to the level when a 1:1 mixture of both RT primers was used (n = 3). *F*, comparison of PTH and PTH-AS expression levels among various cells. Each expression level is shown as a relative value with the value of 1 in the PA sample (PA #1) of PTH-AS expression (n = 3). *G*, PTH expression level in T47D cells with transient overexpression of PTH-AS. All values were normalized to *GAPDH* (n = 3). Data are shown as the mean ± SD. ∗∗*p* < 0.01 by unpaired *t* test. cDNA, complementary DNA; lncRNA, long noncoding RNA; ND, not detected; ns, not significant; PTH, parathyroid hormone; qPCR, quantitative PCR; RACE, rapid amplification of complementary DNA ends.

A novel lncRNA previously discovered in ectopic PTH-producing MFH and PA with PTH hypersecretion was located approximately 3 kbp downstream of the PTH gene (Fig. 1*A*). Given that the 4-1 fragment was amplified only when reverse transcribed with the sense primer RT-4F1 rather than the antisense primer RT-4R, it was determined that the lncRNA of interest was encoded in the sense strand (Fig. 1*B*). Primer walking identified the lncRNA in the range 13,487,670 to 13,490,086 on human chromosome 11. In addition, 5ʹ- and 3ʹ-rapid amplification of complementary DNA (cDNA) ends (RACE) determined the exact location and length of lncRNA transcripts and identified three transcripts with different lengths: 667 bp (13488037–13488703), 820 bp (13488037–13488856), and 826 bp (13488037–13488862) (Figs. 1*C* and S1). The starting points of these transcripts are identical, so they appear to be transcription variants. Taken together, a novel intergenic lncRNA encoded in the sense strand, which is the antisense of the PTH gene, was identified, and we named it “PTH-AS” according to Wright’s nomenclature (17).

Next, we investigated the intracellular distribution, which is a clue to lncRNA function. The amount of PTH-AS in nuclear and cytoplasmic RNA was quantified by real-time quantitative RT–PCR (qRT–PCR). As shown in Figure 1*D*, 93.8% of PTH-AS was present in the nucleus, whereas only 6.2% was present in the cytoplasm, suggesting that PTH-AS is highly enriched in the nucleus. In addition, reverse transcription with oligo (dT)_18_ primers instead of random primers resulted in more efficient PCR amplification of the 4-1 fragment, indicating that PTH-AS was polyadenylated (Fig. 1*E*).

### Ectopic PTH expression cannot be induced by PTH-AS expression alone

PTH-AS expression was detected in one PA case with PTH overexpression in addition to ectopic PTH-producing MFH but not in PTH–nonexpressing cell lines, including some endocrine cancers (Fig. 1*F*). These data implied that PTH-AS was expressed only in tumors expressing PTH, so we suspected that PTH-AS might be involved in the regulation of PTH expression. Presently, there are no transfectable PTH-producing cell lines, so we attempted to elucidate the effect of exogenous PTH-AS on PTH expression using cells that do not express PTH. When an expression vector containing the PTH-AS coding region was transfected into PTH–nonexpressing human breast cancer T47D cells to transiently express PTH-AS, PTH expression was not induced (Fig. 1*G*). Similar results were obtained with other PTH–nonexpressing cells such as human breast cancer MCF7 and human lung cancer A549 (Fig. S2). Therefore, it was considered that PTH-AS expression alone does not induce ectopic PTH gene expression in nonparathyroid cells.

### Effects of PTH-AS expression on gene expression in T47D cells

**Figure 2:**
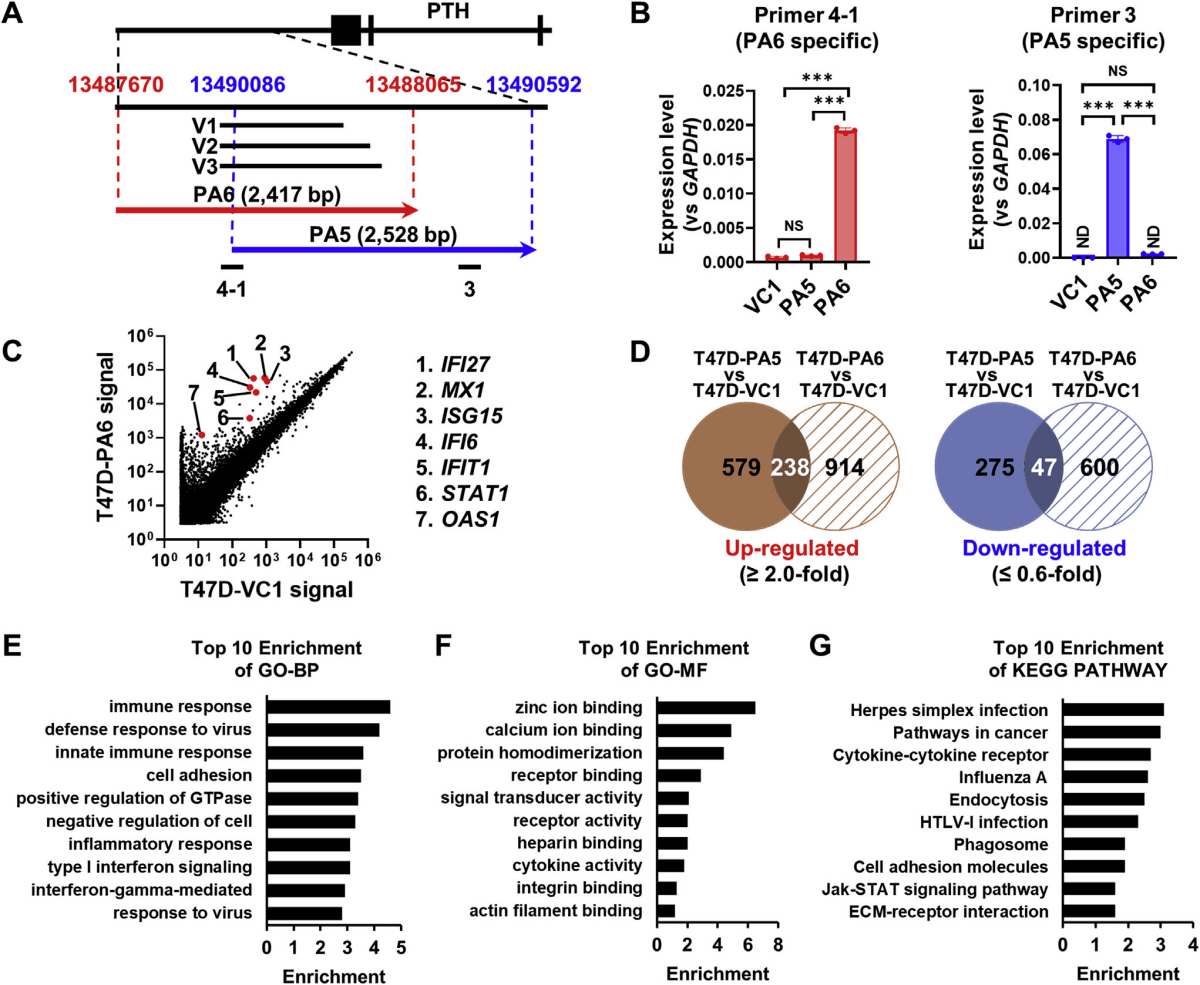
**Effect of PTH-AS expression on gene expression in T47D cells.***A*, the location of the insertion sequence used to generate the PTH-AS expression vector. PA6 (*red arrow*) covers the full length of PTH-AS, whereas PA5 (*blue arrow*) lacks a part of the 5ʹ side of the PTH-AS sequence. Fragments 4-1 and 3 represent PA6- and PA5-specific PCR fragments, respectively. *B*, overexpression of PA5 or PA6 in T47D cells. In qRT–PCR for the detection of PA6-specific fragment 4-1 and PA5-specific fragment 3, each value was normalized to *GAPDH* (n = 3). *C*, scatter plot of gene expression in T47D-VC1 cells and T47D-PA6 cells. The *red plots* show the IRDS gene. *D*, PTH-AS expression–specific genetic alterations. The Venn diagram shows the number of genes with increased or decreased expression levels in T47D-PA5 or T47D-PA6 cells compared with T47D-VC1 cells. The *shaded areas* are PTH-AS expression–specific alterations, and the overlapping areas are nonspecific. *E*–*G*, Gene Ontology (GO) analysis of the bioprocesses (GO-BP) (*E*), molecular functions (GO-MF) (*F*), and KEGG (*G*). Data are shown as the mean ± SD. ∗∗∗*p* < 0.001 by one-way ANOVA. IRDS, interferon-related DNA damage resistance signature; KEGG, Kyoto Encyclopedia of Genes and Genomes; ND, not detected; ns, not significant; PTH, parathyroid hormone; qRT–PCR, quantitative RT–PCR.

In general, the majority of nuclear-localized lncRNAs contribute to chromatin remodeling and RNA processing and are involved in global gene regulation in a variety of ways (2, 18, 19). Therefore, we prepared T47D cells that stably express PTH-AS and comprehensively analyzed the effect of PTH-AS on gene expression. Because the sequence near the PTH-AS gene did not fit the design of the primer for vector construction, we adopted PA6 fragment (chromosome 11, 13487670–13488065, 2417 nt) that are longer than covering the transcriptional region determined by 5′- and 3′-RACE. The common site on the 5′ side of the three PTH-AS transcripts may be functionally important. Therefore, we decided to use a fragment lacking part of the 5′ end of the PTH-AS transcription region as a negative control for this experiment. Moreover, to prevent the fragment size from affecting subsequent experiments, we used PA5 fragment (chromosome 11, 13490086–13490592, 2528 nt) that is similar in size to the PA6 fragment (Fig. 2*A*). To eliminate artifacts from extracellular gene transfer, microarray analysis was performed on T47D cells expressing incomplete PTH-AS (T47D-PA5) in addition to full-length PTH-AS–expressing cells (T47D-PA6) or vector control (T47D-VC1) (Fig. 2*B*). According to our definition (see the Experimental procedures section), 1453 genes were differentially expressed in T47D-PA6 compared with T47D-VC1 (Fig. 2*C*). Excluding genes whose expression was altered in T47D-PA5 cells expressing incomplete PTH-AS, PTH-AS expression in T47D cells upregulated 914 genes and downregulated 600 genes (Fig. 2*D*). GO analysis using the public bioinformatics tool DAVID (https://david.ncifcrf.gov/) classified many of the altered genes as immune-related bioprocesses, including “immune response” (*p* = 2.30E-09), “type I interferon signaling pathway” (*p* = 1.80E-27), and “signal transduction via interferon gamma pathway” (*p* = 7.00E-22) as well as “cell adhesion” (*p* = 7.30E-04) (Fig. 2*E*). Analysis by REVIGO (http://revigo.irb.hr/) revealed that these GO terms build clusters specifically associated with the immune response (Fig. S3). In addition, terms such as “integrin binding” (*p* = 1.00E-03) and “actin filament binding” (*p* = 1.80E-02) were listed as related molecular functions (Fig. 2*F*) as well as terms such as “pathways in cancer” (*p* = 4.10E-03), “cell adhesion molecules” (*p* = 1.20E-05), “extracellular matrix (ECM)–receptor interaction” (*p* = 2.20E-04), and “JAK–STAT signaling pathway” (*p* = 1.50E-03), which are listed as related Kyoto Encyclopedia of Genes and Genomes pathways (Figs. 2*G* and S4–S7).

### STAT1 target IRDS genes were upregulated with PTH-AS expression in T47D cells

**Figure 3:**
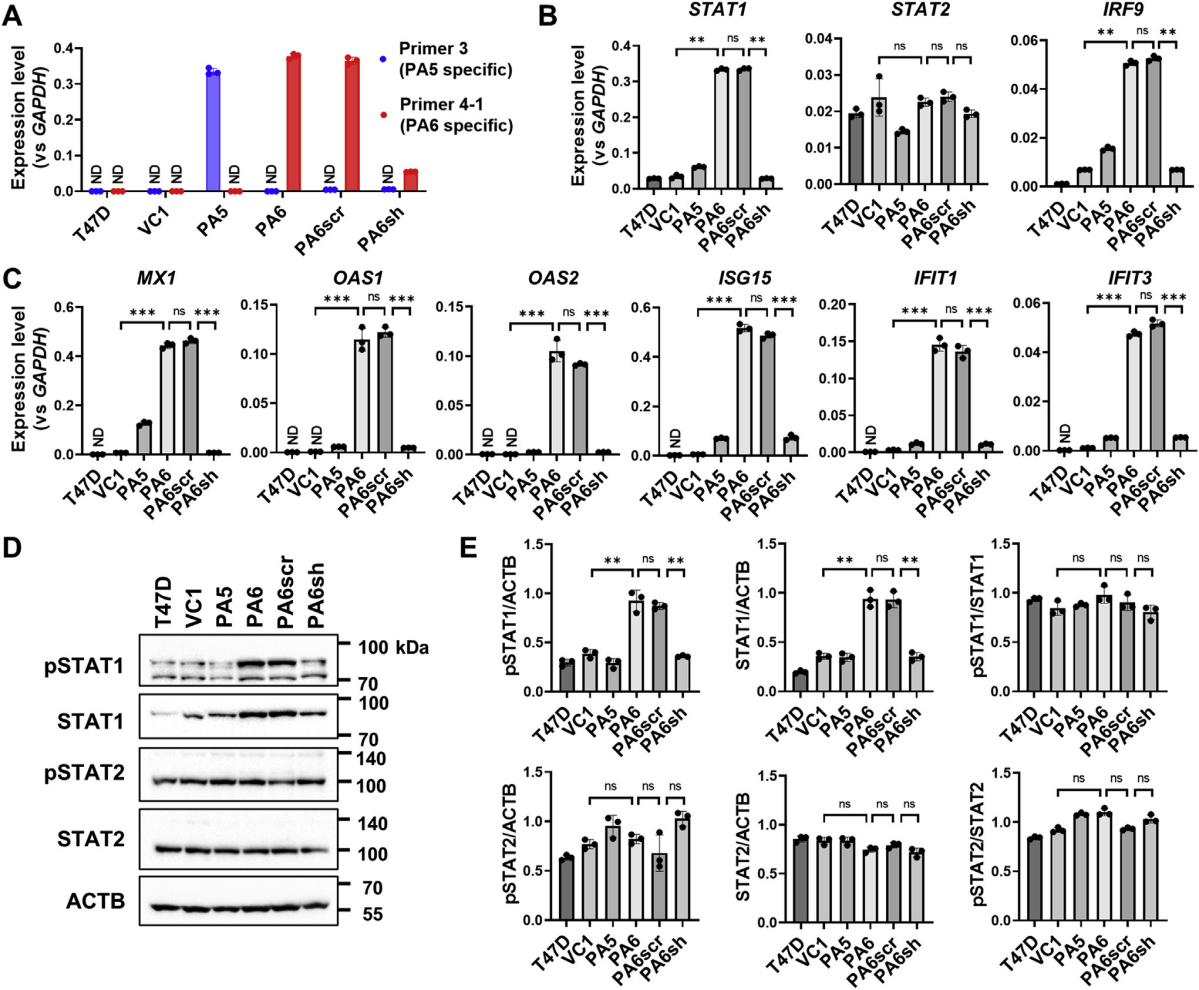
**Upregulation of STAT1 target IRDS genes associated with PTH-AS expression in T47D cells.***A*, stable expression of PTH-AS in T47D cells and its shRNA-mediated knockdown. *B* and *C*, expression levels of JAK–STAT signaling–related genes (*B*) and IRDS genes (*C*) in the cells shown in (*A*). The mRNA level of each gene was quantified by qRT–PCR and normalized by *GAPDH* expression (n = 3). *D* and *E*, detection of phosphorylated STAT1/2 by Western blotting. The uncropped images are shown in Fig. S26. Signal intensity was quantified using ImageJ software and normalized to β-actin (ACTB). The ratio of phosphorylated protein to total protein was also calculated (n = 3) (*E*). Data are shown as the mean ± SD. ∗∗*p* < 0.01 and ∗∗∗*p* < 0.001 by one-way ANOVA. IRDS, interferon-related DNA damage resistance signature; JAK, Janus kinase; ND, not detected; ns, not significant; PTH, parathyroid hormone; qRT–PCR, quantitative RT–PCR; STAT1, signal transducer and activator of transcription 1.

Gene set enrichment analysis also strongly suggested a correlation between PTH-AS expression and JAK–STAT signaling (Fig. S8*A*). Supporting these data, the PScan database (http://159.149.160.88/pscan/) (20) listed STAT1 and IRF9 as potential transcription factors that bind to the promoters of genes whose expression levels were significantly affected by PTH-AS expression in T47D cells (Fig. S8*B*). In fact, our microarray analysis showed the expression of STAT1 and IRF9 as well as their target, ISGs was higher in T47D-PA6 cells than in T47D-VC1 and T47D-PA5 cells (Fig. S9). In particular, the expression levels of genes, such as *IFI6*, *MX1*, *IFIT1*, *IFIT3*, *ISG15*, *OAS1*, and *OAS2*, which belong to the cancer-promoting ISG subset IRDS, were markedly increased (Fig. S9*B*). To further clarify the correlation between expression levels of PTH-AS and IRDS, we designed PTH-AS–specific shRNAs and transfected them into T47D-PA6 cells to suppress exogenous PTH-AS expression (Figs. 3*A* and S10). Subsequent qRT–PCR analysis revealed that STAT1 and IRDS gene expression levels in T47D cells increased with PTH-AS overexpression but decreased with PTH-AS knockdown (Fig. 3, *B* and *C*). PTH-AS overexpression and knockdown experiments in MCF7 and A549 cells also confirmed a positive correlation between PTH-AS and IRDS expression levels (Figs. S11 and S12). These data suggest that PTH-AS is involved in the regulation of STAT1 and its downstream IRDS expression and that it is not limited to specific cells.

Unlike common ISGs, IRDS expression is induced *via* the U-ISGF3 complex containing unphosphorylated STAT1, rather than the ISGF3 complex containing phosphorylated STAT1 (pSTAT1) (15). Western blotting analysis showed that total STAT1 (tSTAT1) protein increased with PTH-AS expression in T47D cells (Fig. 3, *D* and *E*). pSTAT1 showed a similar tendency to tSTAT1, but the pSTAT1/tSTAT1 ratio was not affected by PTH-AS expression, suggesting that STAT1 phosphorylation was not enhanced. STAT2 was unaffected by PTH-AS expression at either total or phosphorylated protein levels (Fig. 3*E*). Therefore, PTH-AS expression may be involved in inducing IRDS expression through increased levels of STAT1 protein rather than phosphorylation of STAT1.

### Effect of PTH-AS expression on cancer hallmarks of T47D cells

**Figure 4:**
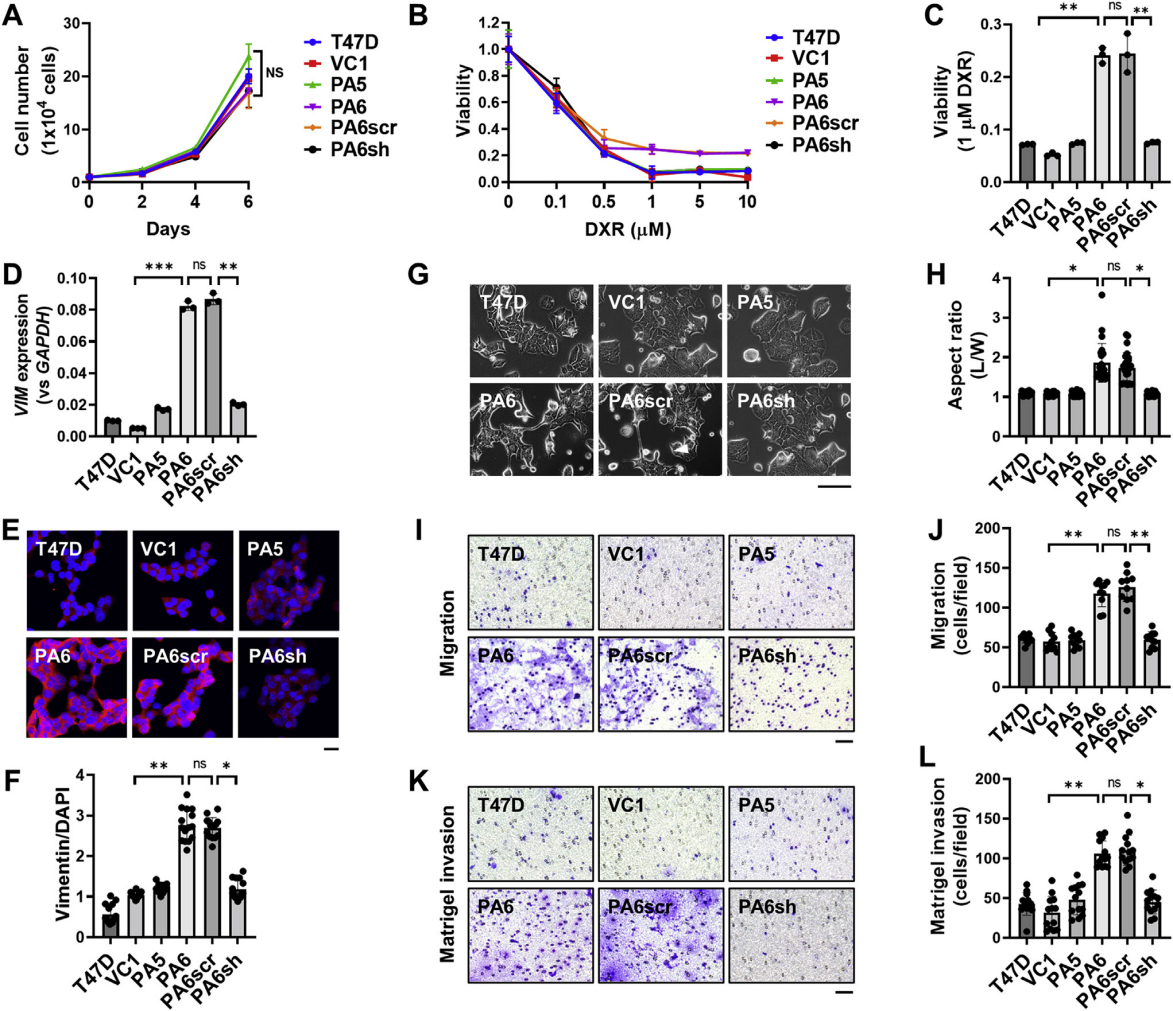
**Effects of PTH-AS expression on malignant properties of T47D cells.***A*, cell proliferation. Cells (1 × 10^4^) were seeded, and the number of viable cells was counted after 2, 4, and 6 days using trypan blue exclusion (n = 3). *B* and *C*, resistance to DXR. Cells (5 × 10^5^) were treated with DXR at the indicated concentrations (*B*) or 1 μM (*C*) for 48 h, and cell viability is assessed by MTT assay. The viability of DXR-untreated cells is shown as 1. *D*, the gene expression level of vimentin was quantified by qRT–PCR and is shown relative to the *GAPDH* expression level (n = 3). *E* and *F*, immunofluorescence analysis of vimentin. Cells were stained with an antivimentin antibody and observed using a confocal laser scanning microscope. The scale bar represents 50 μm (*E*). The fluorescence intensities of vimentin and DAPI were quantified by ImageJ software, and the ratio of vimentin to DAPI was calculated (n = 15) (*F*). *G*, bright field image of cells (200× and 400×). The scale bar represents 50 μm. *H*, cell aspect ratio. The length (L) and width (W) of cells were measured with ImageJ, and the aspect ratio (L/W) was calculated (n = 25). *I* and *J*, migration assay. *K* and *L*, Matrigel invasion assay. After culturing for 12 h for the migration assay and 24 h for the invasion assay, the infiltrating cells were observed in the bright field (100×) (*I* and *K*), and the number of cells per field was counted (n = 15) (*J* and *L*). The scale bars represent 100 μm. Data are shown as the mean ± SD. ∗*p* < 0.05, ∗∗*p* < 0.01, and ∗∗∗*p* < 0.001 by one-way ANOVA. DAPI, 4′,6-diamidino-2-phenylindole; DXR, doxorubicin; MTT, 3-(4,5-dimethylthiazol-2-yl)-2,5-diphenyl-2H-tetrazolium bromide; ns, not significant; PTH, parathyroid hormone; qRT–PCR, quantitative RT–PCR.

IRDS can promote tumor malignancy by causing cancer cells to increase EMT, metastatic potential, and resistance to DNA-damaging agents (14, 15, 21, 22). Therefore, we investigated whether the expression of PTH-AS, which upregulates IRDS, affects the malignant properties of cancer cells. There was no effect of PTH-AS expression on the proliferation of T47D cells (Fig. 4*A*). When treated with the DNA-damaging agent DXR, PTH-AS expression in T47D cells did not affect cell viability at low DXR concentrations (<1 μM) but did lead to a significant drug resistance at high DXR concentrations (≥1 μM) (Fig. 4, *B* and *C*). Of the EMT markers whose expression levels were examined, only the mesenchymal marker vimentin showed a positive correlation with PTH-AS expression (Figs. 4, *D*–*F* and S13). The morphology of T47D cells showed no apparent EMT-like changes with PTH-AS expression but tended to be slightly elongated (Fig. 4, G and *H*). Next, the effect of PTH-AS expression on the motility and invasion of T47D cells was investigated. The results showed that the two-dimensional motility by the wound healing assay was not affected by PTH-AS expression (Fig. S14), but transwell migration (Fig 4, *I* and *J*) and Matrigel invasion were significantly promoted (Fig. 4, *K* and *L*). However, matrix metalloproteinase expression, which is strongly associated with infiltration, was very low in all cells and did not correlate with PTH-AS expression (Fig. S15). The aforementioned GO analysis showed altered expression of genes related to “cell adhesion,” “integrin binding,” and “cell adhesion molecule” with PTH-AS expression in T47D cells (Fig. 2, *E*–*G*). These data suggest that PTH-AS expression affects T47D cell adhesion, but *in vitro* assays showed no effect of PTH-AS expression on ECM adhesion (Fig. S16). Taken together, PTH-AS expression did not affect proliferation, two-dimensional motility, or ECM adhesion but enhanced DNA damage agent resistance, as well as migration and invasion.

**Figure 5:**
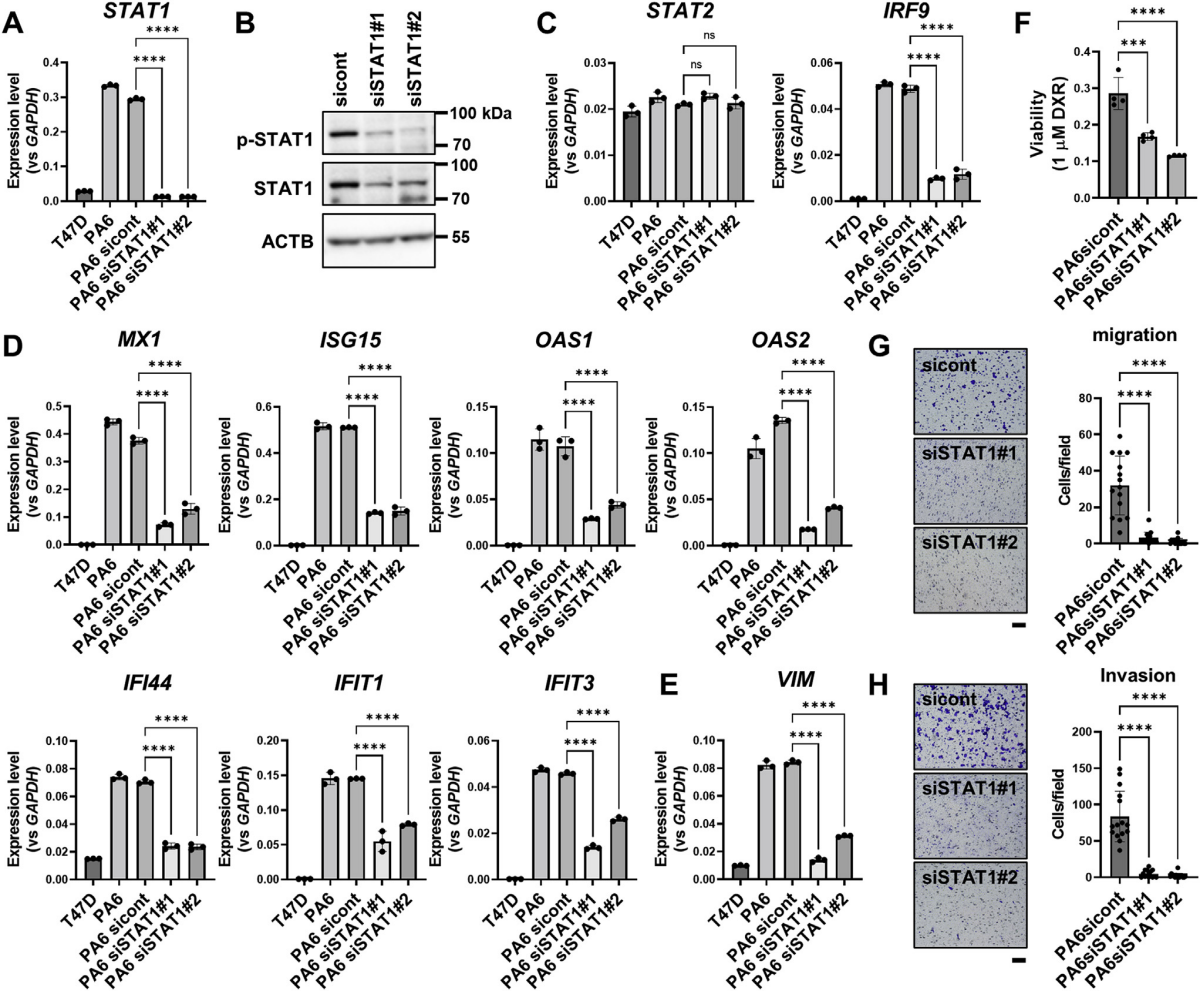
**Effects of STAT1 knockdown on gene expression and cancer characteristics in T47D-PA6 cells.***A* and *B*, confirmation of siRNA-mediated STAT1 knockdown in T47D-PA6 cells by qRT–PCR (n = 3) (*A*) and Western blotting analysis (*B*). The uncropped images are shown in Fig. S26. *C*–*E*, analysis of gene expression levels associated with STAT1 knockdown in T47D-PA6 cells by qRT–PCR (n = 3). mRNA levels of JAK–STAT signaling–related genes (*C*), IRDS genes (*D*), and vimentin (*E*). *F*, resistance to DXR. Each cell (5 × 10^5^) was treated with 1 μM DXR for 48 h, and cell viability was assessed by MTT assay. The DXR-untreated survival rate of each cell was calculated as 1 (n = 4). *G*, transwell migration assay. *H*, Matrigel invasion assay. After culturing for 12 h for the migration assay and 24 h for the invasion assay, the infiltrating cells were stained with crystal violet. The stained cells were observed under a bright field (100×), and the number of cells per field was counted (n = 15). The scale bars represent 100 μm. Data are shown as the mean ± SD. ∗∗∗*p* < 0.001 and ∗∗∗∗*p* < 0.0001 by one-way ANOVA. DXR, doxorubicin; IRDS, interferon-related DNA damage resistance signature; JAK, Janus kinase; MTT, 3-(4,5-dimethylthiazol-2-yl)-2,5-diphenyl-2H-tetrazolium bromide; ns, not significant; qRT–PCR, quantitative RT–PCR; STAT1, signal transducer and activator of transcription 1.

In addition, siRNA-mediated knockdown of STAT1 in T47D-PA6 cells (Fig. 5, *A* and *B*) resulted in reduced expression of IRF9 (Fig. 5*C*), IRDS genes (Fig. 5*D*), and vimentin (Fig. 5*E*). Along with these gene expression changes, the enhancement of DXR resistance (Fig. 5*F*), transwell migration (Fig. 5*G*), and Matrigel invasion (Fig. 5*H*) exhibited by T47D-PA6 cells were canceled. Similar results were obtained in experiments using MCF7 cells and A549 cells as well as T47D cells (Figs. S17–S20). Those imply that PTH-AS expression in T47D cells, MCF7 cells, and A549 cells affects the expression of various genes and cancer properties *via* upregulation of STAT1.

### Expression of PTH-AS did not affect tumor growth of T47D cells but promoted tumor angiogenesis and lung metastasis

**Figure 6:**
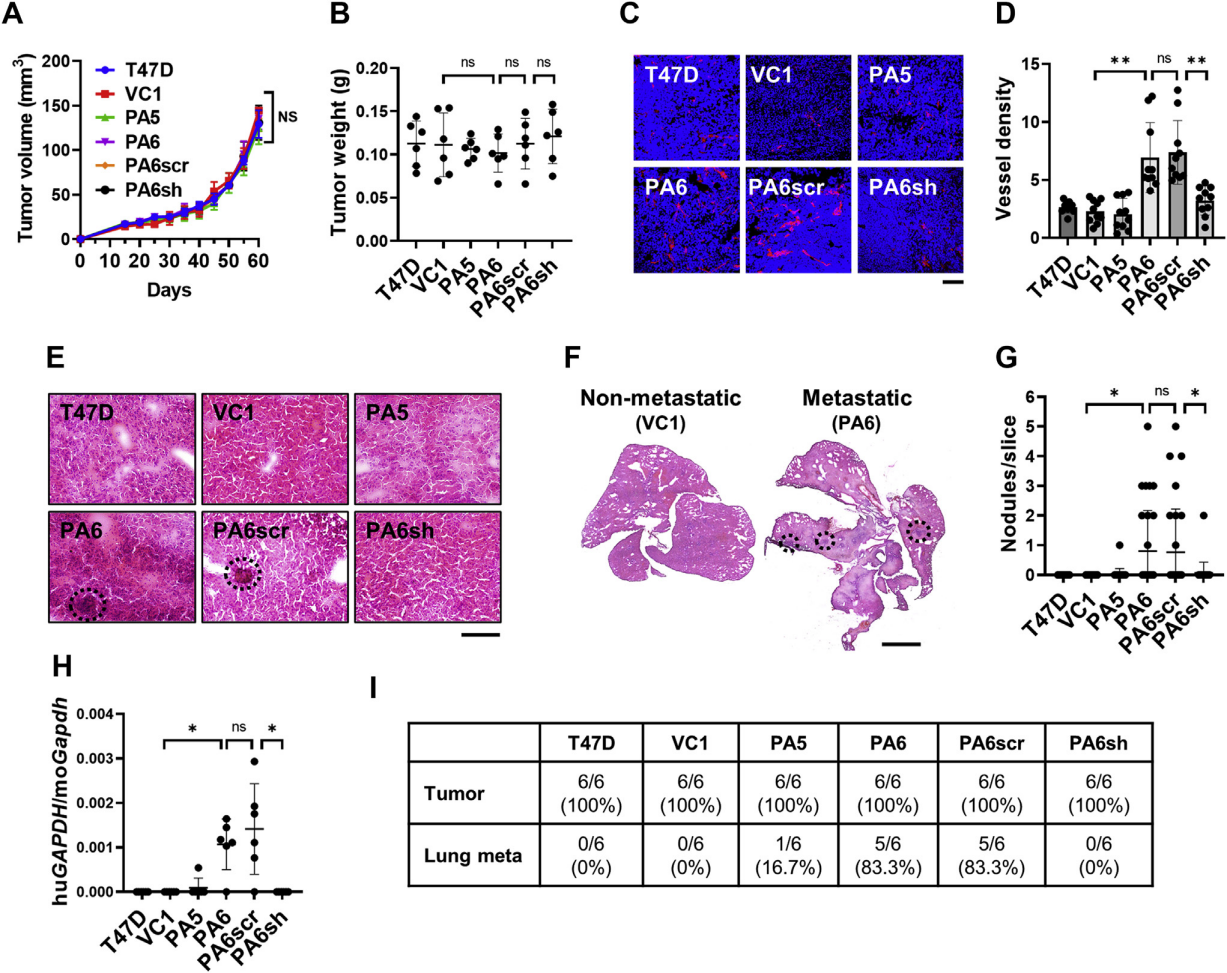
**Effects of PTH-AS expression on T47D tumor growth and lung metastasis.***A*, tumor growth. 1 × 10^7^ cells were subcutaneously transplanted into nude mice. After tumor formation, the tumor size was measured every 5 days, and the volume was calculated (n = 6). *B*, tumor weight. Mice were euthanized 60 days after transplantation to remove the tumor and weighed. *C* and *D*, tumor blood vessel density. Frozen sections of the tumor were fluorescently stained with an antibody against the vascular endothelial cell marker CD31 and observed with a confocal laser scanning microscope (400×) (*C*). Tumor vessel density was calculated using ImageJ software (n = 10) (*D*). The scale bar represents 50 μm. *E* and *F*, HE-stained image of lungs in tumor-bearing mice. Lung sections were observed at high magnification (800×) (*E*) and low magnification (40×) (*F*). The scale bars represent 100 μm and 2 mm, respectively. *G*, micrometastasis. Metastatic nodules (*dotted line*) were counted for each section (n = 30). *H*, evaluation of lung metastasis by qRT–PCR. Using RNA extracted from the lungs of tumor-bearing mice, the ratio of the expression level of human *GAPDH* derived from transplanted cells to host-derived mouse *Gapdh* was calculated by qRT–PCR (n = 6). *I*, tumorigenicity and lung metastatic rate. Data are shown as the mean ± SD. ∗*p* < 0.05 and ∗∗*p* < 0.01 by one-way ANOVA. ns, not significant; PTH, parathyroid hormone; qRT–PCR, quantitative RT–PCR.

Next, we examined the effects of PTH-AS on tumorigenesis and malignant progression of T47D cells *in vivo*. When cells with different PTH-AS expression levels were subcutaneously transplanted into nude mice and tumor growth was compared, there was no difference in tumor growth rate and tumor weight among them (Fig. 6, *A* and *B*). However, immunofluorescent staining of the vascular endothelial cell marker CD31 showed significantly higher vascular density in tumors derived from PTH-AS–expressing cells, suggesting that PTH-AS expression promoted tumor angiogenesis (Fig. 6, *C* and *D*).

Lung metastases were also evaluated because increased tumor angiogenesis may lead to enhanced hematogenous metastases. Although no gross metastatic nodules were observed on the lung surface of any tumor-bearing mice, more micrometastatic nodules were detected in HE-stained sections of the lungs from mice bearing PTH-AS–expressing tumors (Fig. 6, *E*–*G*). Consistent with these data, the ratio of transplanted cell–derived human *GAPDH* to host-derived mouse *Gapdh* in lung tissue was significantly higher in mice with PTH-AS–expressing tumors (Fig. 6*H*). In our human breast cancer T47D xenograft model, tumorigenesis was observed in all cases regardless of the PTH-AS expression levels in the transplanted cells, but lung metastases were detected more frequently in mice with PTH-AS–expressing tumors (Fig. 6*I*).

Overall, our results demonstrate that PTH-AS expression in T47D cells did not affect tumor formation and growth but did promote tumor angiogenesis and lung metastasis. Similar results were also shown by transplantation experiments using MCF7 cells and A549 cells with various PTH-AS expression levels (Figs. S21 and S22).

### Expression of PTH-AS in T47D cells enhanced macrophage accumulation in tumor tissue

**Figure 7:**
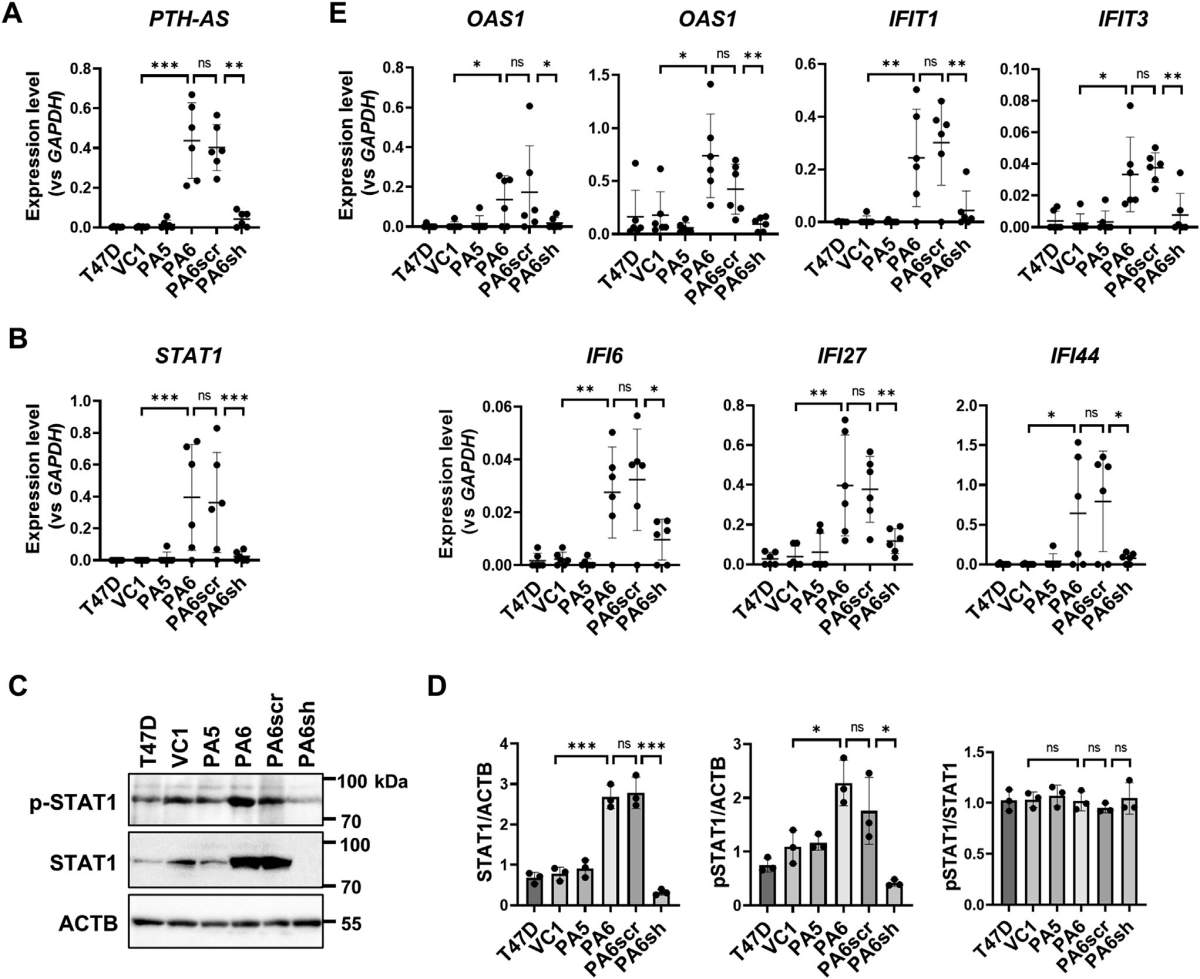
**IRDS expression associated with PTH-AS expression in T47D tumors.***A*, PTH-AS expression levels in tumors. PTH-AS mRNA levels were examined by qRT–PCR using RNAs extracted from the tumors. For normalization, *GAPDH* primers common to human and mice were used (n = 6). *B*, STAT1 expression levels in tumors. STAT1 mRNA levels were examined by qRT–PCR as in (*A*) (n = 6). *C* and *D*, phosphorylation levels of STAT1 in tumors. STAT1 and p-STAT1 were detected by Western blotting using proteins extracted from the tumor (*C*). The uncropped images are shown in Fig. S26. All the antibodies used were human specific and had no crossreactivity to mice. The signal intensity was quantified by ImageJ, and the ratio of STAT and p-STAT1 was calculated (n = 3) (*D*). *E*, expression levels of IRDS genes in tumors. Each mRNA level was examined by qRT–PCR as in (*A*) (n = 6). Data are shown as the mean ± SD. ∗*p* < 0.05, ∗∗*p* < 0.01, and ∗∗∗*p* < 0.001 by one-way ANOVA. IRDS, interferon-related DNA damage resistance signature; ns, not significant; p-↓STAT1, phosphorylated STAT1; PTH, parathyroid hormone; qRT–PCR, quantitative RT–PCR; STAT1, signal transducer and activator of transcription 1.

**Figure 8:**
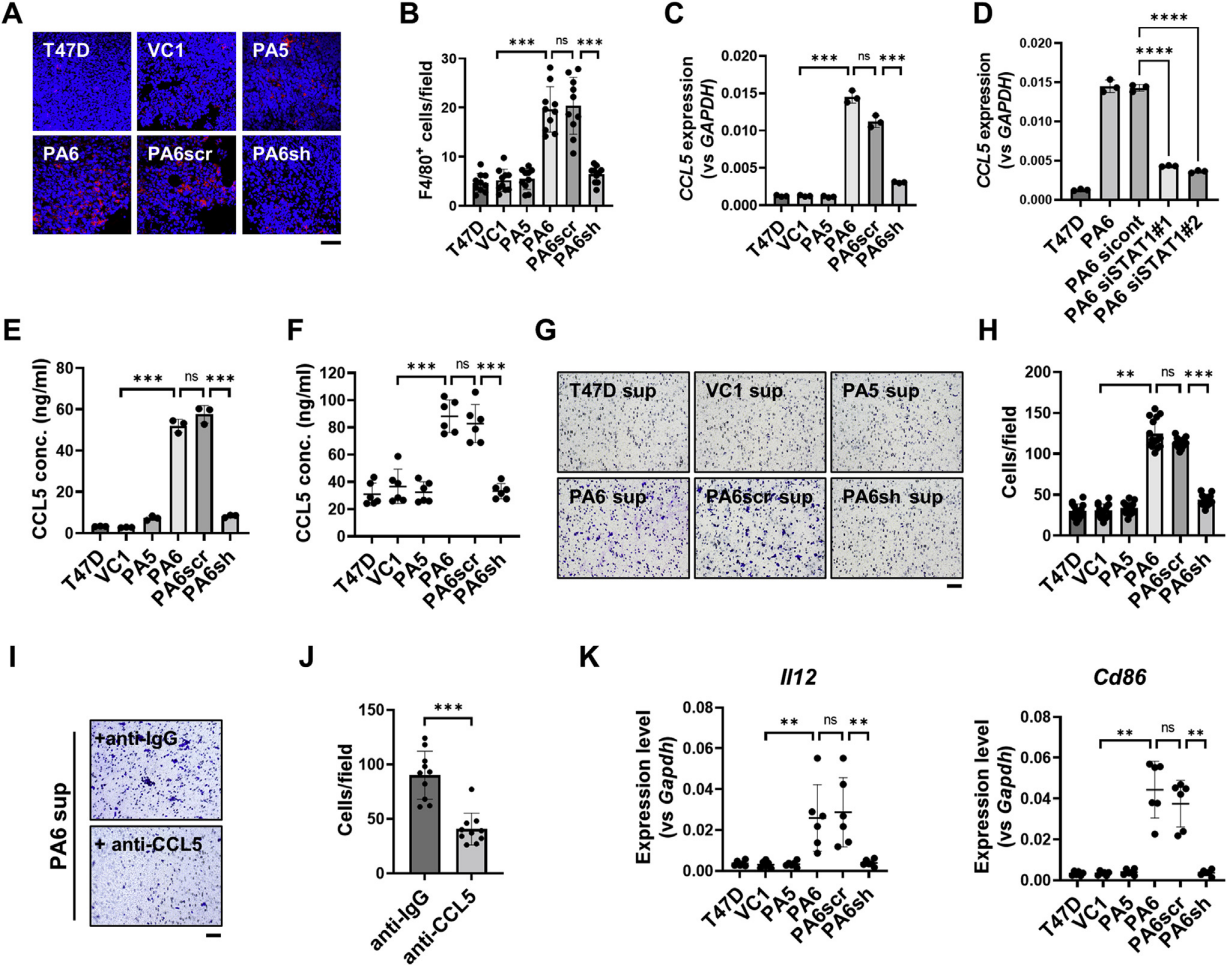
**Effect of PTH-AS expression in T47D tumors on macrophage migration and inflammatory environment in xenografts.***A* and *B*, tumor infiltration of macrophages. Tumor cryostat sections were immunofluorescent stained with anti-F4/80 antibody (*A*), and the number of F4/80-positive cells per field was counted (*B*). The scale bar represents 50 μm. *C*, CCL5 mRNA levels associated with PTH-AS expression in T47D cells (n = 3). *D*, CCL5 mRNA levels in T47D-PA6 cells with STAT1 knockdown (n = 3). *E* and *F*, CCL5 ELISA. The CCL5 protein concentrations in the culture supernatants (n = 3) (*E*) and in the tumor extracts (*F*) were measured by ELISA (n = 6). *G* and *H*, effect of PTH-AS expression on T47D cells of macrophage migration. A chemotaxis assay was performed using mouse macrophage RAW264.7 cells and culture supernatants of T47D cells with different PTH-AS expression levels (*G*). After 12 h, the migrated RAW264.7 cells were observed in a bright field (200×), and the number of cells per field was counted (n = 15) (*H*). *I* and *J*, CCL5-dependent macrophage chemotaxis. Anti-IgG antibody (negative control) or anti-CCL5 antibody was added to the culture supernatant of T47D-PA6 cells, and the migration of RAW264.7 cells was evaluated by the same method as (*G*) and (*H*). *K*, expression levels of M1 markers in xenografts. The expression levels of each gene were examined by qRT–PCR using RNA extracted from the tumor (n = 6). The expression levels of the M2 markers are shown in Fig. S24. For normalization, *GAPDH* primers common to human and mice were used. Data are shown as the mean ± SD. ∗∗*p* < 0.01 and ∗∗∗*p* < 0.001 by one-way ANOVA. IgG, immunoglobulin G; ns, not significant; PTH, parathyroid hormone; qRT–PCR, quantitative RT–PCR; STAT1, signal transducer and activator of transcription 1.

Next, we examined the effect of PTH-AS expression in cancer cells on the tumor microenvironment. PTH-AS expression from transplanted tumor cells was reflected in xenografts (Fig. 7*A*). Tumors with high PTH-AS expression maintained high levels of human STAT1 mRNA and protein from transplanted cells (Fig. 7, *B*–*D*). Concomitantly, PTH-AS–expressing tumors also showed high expression of a series of IRDS genes (Fig. 7*E*). Recent reports have demonstrated that high STAT1 mRNA levels in breast cancer tissues are associated with macrophage infiltration and poor prognosis (23). Consistent with this, immunohistochemical analysis using an antibody against the macrophage marker F4/80 revealed that more macrophages were accumulated in PTH-AS–expressing tumors with high STAT1 expression (Fig. 8, *A* and *B*). Macrophages in tumor tissue, so-called tumor-associated macrophages (TAMs), as well as tumor cells are the major sources of vascular endothelial growth factor (VEGF) and induce tumor angiogenesis. There was no correlation between VEGF expression and PTH-AS expression levels in cultured cells (Fig. S23*A*). In tumors, the expression level of human VEGF derived from transplanted cells was very low and was not affected by PTH-AS expression. In contrast, recipient-derived mouse VEGF mRNA and protein levels were significantly increased in tumors with high PTH-AS expression (Fig. S23, *B* and *C*). These results suggest that VEGF produced primarily by recipient-derived tumor stromal cells, presumably TAM, rather than transplanted tumor cells, is involved in enhancing angiogenesis in high PTH-expressing tumors.

Finally, we investigated the factors involved in TAM recruitment associated with PTH-AS expression. Microarray and qRT–PCR data showed that PTH-AS expression in T47D cells increased the gene expression of CCL5, a chemokine that promotes macrophage recruitment (Fig. 8*C*). CCL5 is known to be one of the STAT1 target genes (24), and it was confirmed that the increased expression of CCL5 in T47D-PA6 cells was canceled by STAT1 knockdown (Fig. 8*D*). ELISA analysis revealed that the CCL5 concentration in the culture supernatant was higher in T47D cells with high PTH-AS expression than in those with low PTH-AS expression (Fig. 8*E*). CCL5 levels in protein extracts from T47D tumors with high PTH-AS expression were also high (Fig. 8*F*). Next, a chemotactic assay was performed using mouse macrophage RAW264.7 cells to determine whether T47D cells with high PTH-AS expression promote macrophage infiltration *via* CCL5 secretion. The results showed that chemotactic migration of RAW264.7 cells was promoted by the culture supernatant of cells with high PTH-AS expression (Fig. 8, *G* and *H*) and attenuated by the addition of CCL5-blocking antibody (Fig. 8, *I* and *J*). In addition, CCL5 is associated with M1 polarization (25). According to qRT–PCR analysis, the expression of mouse M1 macrophage markers such as *Il12* and *Cd86* was increased in tumors with high PTH-AS expression (Fig. 8*K*), but the expression of M2 markers such as *Il10*, *Cd163*, and *Arg1* was not changed (Fig. S24).

Taken together, PTH-AS expression may increase CCL5 expression downstream of STAT1 and contribute to macrophage recruitment into tumors and changes in the microenvironment of inflammatory tumors.

## Discussion

Over the last decade, lncRNAs have received increasing attention because they have been shown to be involved in important biological processes and various diseases. Recent advances in analytical technology have led to the discovery of new lncRNAs, but their annotations are often inadequate. The purpose of this study was to clarify the nature and function of the novel lncRNA downstream of the PTH gene detected in ectopic PTH-producing MFH tumors. Herein, we demonstrated that the lncRNA of interest in our study is an intergenic lncRNA encoded by the antisense strand in the downstream region of the PTH gene, and we named it PTH-AS. Forced but transient expression of PTH-AS in PTH–nonexpressing T47D breast cancer cells did not induce PTH expression and upregulated STAT1 and its downstream IRDS genes. *In vitro* and *in vivo* analyses revealed that PTH-AS expression increased the malignant properties of cancer cells and promoted metastasis but not tumor growth. In addition, STAT1 knockdown experiments showed that PTH-AS regulates cancer characteristics along with the expression of various genes, including IRDS, through STAT1 upregulation.

In T47D cells, STAT1 was upregulated with PTH-AS expression, inducing the expression of the target gene for JAK–STAT signaling, such as ISGs. Notably, there was a significant increase in the expression of the cancer-promoting IRDS, an ISG subgroup, compared with the common antitumor ISGs (13, 14, 22). In addition to promoting EMT and invasion, IRDS expression in cancer cells is particularly strongly associated with resistance to DNA damage induced by antitumor agents and radiation (15). Indeed, PTH-AS–expressing T47D cells showed resistance to the DNA-damaging drug DXR, suggesting that PTH-AS has great potential to contribute to chemoresistance through IRDS upregulation. IRDS expression levels in a variety of cancer tissues, including breast cancer, strongly correlate with resistance to chemotherapy and radiation therapy and are expected to be useful as clinical markers for prognosis and diagnosis (15). Therefore, PTH-AS, which regulates IRDS expression, may also be a promising biomarker, although further validation in various cancers is needed.

IRDS also can increase metastasis and poor outcome by promoting TAM recruitment and suppressing T-cell cytotoxicity through tumor–stromal interactions in tumor microenvironments (21, 22). In our xenograft model, PTH-AS expression did not affect tumor growth but instead stimulated lung metastases. In addition, PTH-AS–expressing T47D tumors significantly increased TAM infiltration and possibly TAM-derived mouse VEGF production, along with potent tumor angiogenesis. Regarding the increased TAM infiltration in tumors with high PTH-AS expression, it is suspected that the increased expression of CCL5, which is the target gene of STAT1, is involved. In fact, an *in vitro* chemotaxis assay confirmed that cells with high PTH-AS expression promote CCL5-mediated migration of mouse macrophages (Fig. 8, *G*–*J*). In addition, tumors with high PTH-AS expression tended to enhance the M1 phenotype (Figs. 8*K* and S24), which is strongly associated with CCL5. Originally, the M1 phenotype was considered to be a sign of good prognosis because it induces antitumor activity. However, in recent years, this idea has been questioned, and evidence has been reported that M1 macrophages also promote malignant progression in various cancers, including breast cancer (26). Given these findings, PTH-AS expression in tumor cells may induce TAM infiltration and M1 polarization through increased STAT1-induced CCL5 production, creating a tumor microenvironment prone to malignant progression.

Unfortunately, our study does not explain how PTH-AS regulates STAT1 expression. We investigated the expression levels of several miRNAs involved in STAT1 expression regulation, but none were correlated with PTH-AS expression levels. Therefore, it was not possible to clarify whether PTH-AS regulates STAT1 expression *via* miRNA. To date, there have been several reports of nuclear or cytoplasmic lncRNAs involved in the expression of STAT1 (27, 28). Among them, unlike general nuclear-localized lncRNAs that directly regulate gene expression as part of the ribonuclear protein complex (19, 29), type 1 diabetes–associated lnc13 translocates from the nucleus to the cytoplasm and indirectly regulates STAT1 expression through interaction with other biomolecules (30). This indicates that even in the case of nuclear-localized lncRNAs such as PTH-AS, its regulatory mechanism of STAT1 expression needs to be considered both directly and indirectly. Interestingly, many lncRNAs have recently been reported that promote tumor malignancies in a variety of cancers through the production of short peptides (31, 32, 33). Figure 1*D* shows that PTH-AS is distributed almost in the nucleus. Nuclear-localized lncRNAs often have untranslated transcripts (34), but these data do not completely rule out the possibility that PTH-AS will produce short peptides. The coding potential of lncRNA can be investigated to some extent by conservative and translational omics analysis, as well as predictions of ORFs, translational starter elements, and m6A modifications, using genetic information (32). Analysis using the ORF finder (https://www.ncbi.nlm.nih.gov/orffinder/) identified three promising ORF candidates based on the PTH-AS sequence. However, all these ORFs are presumed unlikely to have coding potential because of the lack of evolutionary conservation and homology with known protein domains and the inability to template significant protein production. In fact, it was concluded that well-known lncRNAs such as *XIST* and *H19* have no coding potential for these reasons (35, 36, 37). However, evidence that PTH-AS has no coding potential is merely a computational prediction, so reliable experimental evidence may need to be provided by ribosome profiling. Therefore, at this time, it is necessary to research the details of the PTH-AS function without excluding the possibility of coding.

LncRNAs tune tissue-specific gene expression, and some regulate the expression of adjacent genes in *cis* (18, 38, 39, 40, 41, 42, 43). Therefore, we initially assumed that PTH-AS was one of the key determinants of the expression of the nearby PTH gene, and that its unexpected expression in PTH–nonexpressing cells violated the parathyroid specificity of PTH output, resulting in the ectopy of PTH expression. To date, the mechanism (s) by which the PTH gene is exclusively expressed in a parathyroid-specific manner remains almost completely unknown. This is in a stark contrast to the situation in the PTH-related peptide gene, which is a functional ancestral counterpart of PTH (44). The PTH-related peptide gene is frequently produced in a manner almost free from the constraint of tissue-specific expression, especially in advanced humoral hypercalcemia of malignancy (45, 46). In contrast, ectopic PTH-producing tumors have been reported in only a few cases, including the MFH we analyzed (47, 48, 49, 50, 51, 52, 53, 54, 55, 56, 57, 58, 59, 60, 61, 62, 63). This extreme rarity may reflect the imposition of strict restrictions on parathyroid-specific PTH expression. Unfortunately, contrary to our expectations, non–PTH-expressing cells did not express PTH by forced expression of PTH-AS, so we concluded that PTH-AS expression alone cannot induce PTH expression, at least in these cultured cells. However, it cannot be ruled out that PTH-AS may regulate PTH expression only in cooperation with abnormalities in other factors such as calcium-sensing receptor and cytochrome P450 family 24 subfamily A member 1, which we postulated may be potential regulators of ectopic PTH production (12). Therefore, this possibility should continue to be considered.

Abnormal expression of lncRNA is often because of aberrant chromatin status (64). Consistent with this, chromatin remodeling is expected to be involved in the expression of PTH-AS. Supporting this, frameshift mutations causing chromatin-remodeled α-thalassemia/mental retardation syndrome X-thalassemia deficiency have been detected in an ectopic PTH-producing MFH tumors with PTH-AS expression (12). α-thalassemia/mental retardation syndrome X-thalassemia deficiency alters the expression of various genes through both genomic instability and transcriptional shift (65), which may be a potential cause of PTH-AS expression.

In this study, human breast cancer T47D cells were used mainly to reveal some of the functions of PTH-AS. Originally, PTH-AS was detected in PTH-overexpressing PA together with ectopic PTH-producing MFH, so it may be involved in the development of PA and associated hyperparathyroidism in ways other than those described here. However, there were no functional parathyroid cells or PA-derived clonal cell lines under culture conditions, making this problem difficult to address. Recently, Lawton *et al.* (66) demonstrated an attractive culture protocol that can reproduce functional parathyroid cells. Using this new technology, another function of PTH-AS related to PA progression may be revealed.

In summary, our study revealed a couple of the properties and functions of PTH-AS, a novel lncRNA that we previously discovered in ectopic PTH-producing MFH. Although the exact molecular mechanism has not yet been clarified, it was found that PTH-AS expression in T47D cells upregulates STAT1 and its downstream cancer-promoting IRDS and CCL5, resulting in enhanced tumor malignancies (Fig. S25). However, this study is limited to lncRNA expression in cancer cell lines that do not express PTH-AS. In the future, it is necessary to explore the expression of PTH-AS in malignant tumors and broadly discuss its clinical role.

## Experimental procedures

### Cells and cell culture

Human breast cancer T47D cells and MCF7 cells and human lung cancer A549 cells were purchased from American Type Culture Collection. All cells were cultured in Dulbecco’s modified Eagle's medium (DMEM) containing 10% fetal bovine serum and 40 μg/ml gentamicin under humid conditions with 21% O_2_ and 5% CO_2_.

### RNA preparation and qRT–PCR

Total RNA was extracted from cells or tissues by the phenol chloroform method using RNAiso (Takara Bio, Inc). Using the PrimeScript RT reagent kit (Takara Bio, Inc), cDNA was synthesized from 1 μg of total RNA after removal of genomic DNA. Random primers were used for reverse transcription reactions other than strand specific. qPCR was performed using Thermal Cycler Dice Real Time System III (Takara Bio, Inc) according to recommended cycling parameters using 1 μl cDNA diluted fivefold with EASY Dilution (Takara Bio, Inc) and TB Green Premix Ex Taq TMII (Takara Bio, Inc). Gene expression levels were normalized to *GAPDH* by the ΔΔCT method. Primer information for chain-specific RT and primer walking, and for other qRT–PCR, is detailed in Tables S1 and S2, respectively.

### Western blot analysis

Cell pellets or homogenized tumors were suspended in radioimmunoprecipitation assay buffer containing the phosphatase inhibitor cocktail PhosSTOP (MilliporeSigma). After centrifugation, the supernatant was subjected to SDS-PAGE. Each 40 μg protein was electrophoresed on a 5 to 20% gradient SDS gel and transferred to a polyvinylidene difluoride membrane (MilliporeSigma) using a semidry blotting device. Membranes were blocked with 5% bovine serum albumin (BSA) and then reacted with horseradish peroxidase–labeled secondary antibody following the primary antibody, as shown in Table S3. Immunoreactive bands were detected by the ChemiDoc Imaging System (Bio-Rad Laboratories, Inc) using Western BLoT Hyper HRP (Horseradish Peroxidase) Substrate (Takara Bio, Inc) and quantified by image analysis software ImageJ (National Institutes of Health).

### 5ʹand 3ʹ-RACE–PCR

To detect the 5ʹ and 3ʹ ends of PTH-AS, the SMARTer RACE 5ʹ/3ʹ Kit (Clontech) was used according to the manufacturer’s instructions. In this method, cDNA was synthesized from polyA^+^ RNA by SMARTer technology, followed by RACE–PCR. 5ʹ- and 3ʹ-RACE products were ligated to pUC19 vector using the In-Fusion HD Cloning Kit (Clontech) and amplified in Stellar Competent Cells (Clontech). The RACE products were sequenced, and their sites were determined by BLAST searches.

### Fractionation of subcellular RNA

The RNA subcellular isolation kit (Active Motif) was used according to the manufacturer’s instructions to isolate cytoplasmic and nuclear RNA. In subsequent qRT–PCR, H19 and MALAT1 were used as positive controls for cytoplasmic and nuclear lncRNAs, respectively.

### Microarray analysis

Total RNA for microarray was prepared using the RNeasy Plus Mini Kit (Qiagen Benelux B.V.). Briefly, 50 ng of total RNA labeled using the Agilent Low-Input Quick Amp Labeling Kit, one color (Agilent Technologies) was hybridized using the SurePrint G3 Human Gene Expression v3 8×60K Microarray Kit (Agilent Technologies). The hybridized microarray slides were scanned using an Agilent scanner. The relative hybridization intensities and background hybridization values were calculated using Feature Extraction Software, version 9.5.1.1 (Agilent Technologies). The data were quantified using feature extraction software (Agilent Technologies) and normalized by the quantile method using statistical processing software R, version 4.0.5 (R Foundation for Statistical Computing). When the expression signals were more than twofold or less than 0.6-fold compared with the control, they were defined as upregulation and downregulation, respectively. Based on these data, GO analysis and clustering were performed using the public databases DAVID (https://david.ncifcrf.gov/) and REVIGO (http://revigo.irb.hr/), respectively.

### Immunocytochemistry

Cells cultured on glass coverslips were fixed with 4% paraformaldehyde and permeabilized with 0.2% Triton X-100. After blocking in 10% goat serum, the cells were reacted with an antivimentin antibody followed by Alexa Fluor 594–labeled secondary antibody, as detailed in Table S3. After nuclear counterstaining with 10 μg/ml 4′,6-diamidino-2-phenylindole (Dojindo), the coverslips were mounted on microscope slides using Dako fluorescent mounting medium (Dako UK Ltd). Cells were observed using a Nikon A1si confocal laser scanning microscope (Nikon).

### Wound healing assay

Artificial wounds were formed by scratching a confluent cell monolayer formed by cells cultured in a 12-well plate. Images were captured at 0, 12, and 24 h, and the width of the gap of each wound was measured using ImageJ software.

### Migration and matrigel invasion assay

Migration and invasion were evaluated using the transwell assay. In the invasion assay, the transwell membrane was coated with 50-fold diluted Matrigel (Corning, Inc). Cells suspended in serum-free DMEM were seeded in the upper chamber of a Chemotaxicell transwell insert (pore size 8 μm; Kurabo), and the lower chamber was filled with DMEM containing 10% fetal bovine serum. After 24 h of incubation, cells that passed through the transwell membrane were fixed with methanol and stained with 0.4% crystal violet. Images were taken using an IX71 inverted microscope (Olympus), and the number of cells was counted.

### Macrophage chemotaxis assays

Macrophage chemotaxis assays were performed using transwell chambers (24-well plate, 8 μm pore size; Corning) and mouse macrophage RAW 264.7 cells. First, to prepare a culture supernatant of cells expressing or lacking PTH-AS, each well (1 × 10^6^ cells) was seeded in 3 ml of serum-free medium, and the culture supernatant was collected 2 days later. For the chemotaxis assay, 2 × 10^5^ RAW264.7 macrophages were placed in each upper chamber, and cell supernatants with or without 1 μg/ml of CCL5 neutralizing antibodies or control immunoglobulin G were placed in the lower chamber. After 4 h, the migrating cells were fixed with formalin and stained with 0.4% crystal violet. For each chamber, five nonoverlapping fields were selected under the microscope and cells were counted.

## ELISA

Human and mouse VEGF protein concentrations in culture supernatant or tumor tissue were quantified using Human VEGF_165_ ELISA Kit (Diaclone) and Mouse VEGF ELISA kit (Bioss Antibodies, Inc) respectively, according to the manufacturer’s protocol. The cell culture supernatant was collected after culturing 1 × 10^6^ cells on a 12-well plate in 1 ml of serum-free medium for 24 h. The tumor protein solution was extracted by homogenizing with 10 μl of radioimmunoprecipitation assay buffer per 10 μg of tumor tissue. Human CCL5 was also measured in the same manner using the LEGEND MAX Human CCL5 (RANTES) ELISA Kit (BioLegend). VEGF concentrations in culture supernatant and tumor protein extract were normalized to cell number and total protein concentration, respectively.

### Overexpression and knockdown experiments

Full-length or incomplete PTH-AS cDNA was cloned into pTARGET mammalian expression vector (Promega) by TA cloning to construct PA6 and PA5 vectors, respectively. In addition, PTH-AS-specific shRNA was designed using the shRNA target design tool provided by Vector Builder. Three types of shRNA expression plasmid vectors (#1, #2, and #3), which were expected to have the highest knockdown score, were obtained. The target sequence for each shRNA is as follows: #1-CCTCGTCAAGGTGACATTTAA, #2-AGGCC TTCAAAGCAGATTAAT, and #3-TTTACAGAGAT GGCAATTAT. These vectors, including vector controls, were transfected into a variety of cells using Lipofectamine 3000 (Invitrogen) according to the manufacturer’s protocol. Stable clones were established by selection with G418 or puromycin, and desirable expression was confirmed by qRT–PCR. Information on the resulting clones is shown in Fig. S9. Knockdown of STAT1 in PTH-AS–expressing cells was performed using commercially available siRNA. The STAT1 siRNA (Thermo Fisher Scientific; Silencer Select Validated siRNA #s278 or #s279) was transfected into the cells of interest according to the product manual of Lipofectamine RNAiMAX Transfection Reagent (Invitrogen). Post-transfection cells were confirmed by qRT–PCR to have reduced STAT1 expression on day 3 and were used in various assays by day 5.

### 3-(4,5-Dimethylthiazol-2-yl)-2,5-diphenyl-2H-tetrazolium bromide assay

Cell viability was determined by 3-(4,5-dimethylthiazol-2-yl)-2,5-diphenyl-2H-tetrazolium bromide (MTT) assay. Cells were seeded at 1 × 10^4^ cells per well in a 96-well plate and treated with various concentrations of DXR (Selleckchem). After 48 h of culture, 3-(4,5-dimethylthiazol-2-yl)-2,5-diphenyl-2H-tetrazolium bromide reagent (Nacalai Tesque) was added to the cells at 0.1 mg/ml. Formazan formed during the 4 h of incubation was dissolved in dimethyl sulfoxide, and the absorbance was measured at 570 nm using a Multiskan GO multiplate reader (Thermo Fisher Scientific).

### Tumor transplantation

All animal studies were conducted in accordance with institutional guidelines for animal care and use. The protocol was approved by the Teikyo University Animal Experiment Ethics Committee (approval no: 19-023). First, 1 × 10^7^ cells suspended in 100 μl serum-free DMEM containing 50% Matrigel were subcutaneously transplanted into 6-week-old female nude mice (BALB/c^nu−/nu−^, six animals per group). Then, the minor axis (a, mm) and major axis (b, mm) of the tumors were measured with a caliper every 3 to 5 days from the 15th day when the tumor was palpable, and the tumor volume (V, mm^3^) was calculated as follows, V = a^2^b. Mice were euthanized 60 days after transplantation. Excised tumors were embedded in Tissue-Tek optimum cutting temperature compound (Sakura Finetek) and frozen.

### Detection of lung micrometastasis

Mouse lung micrometastases were evaluated by human-specific *GAPDH* detection by qRT–PCR. This method correlates with histological analysis and can quantify human cells seeded in a mouse xenograft model with high sensitivity (67). RNA was extracted from finely minced frozen mouse lung pieces using the RNeasy Tissue Kit (Qiagen), and cDNA was synthesized as described previously. Subsequent qRT–PCR was performed using human *GAPDH*-specific or mouse *Gapdh*-specific primers. Next, the human expression level was normalized to the mouse expression level and used as the relative amount of human metastatic cells in mouse lung tissue.

### Immunohistochemical analysis

Frozen sections (10 μm thick) prepared using the cryostat Leica CM3050S (Leica) were fixed with 4% paraformaldehyde and treated with 3% H_2_O_2_/methanol. Sections were blocked with PBS containing 5% normal goat serum, 20% milk powder, and 1% BSA, and reacted with primary antibody against CD31 or F4/80, followed by an Alexa Fluor 594–labelled secondary antibody (Table S3). After nuclear counterstaining, images were captured using a Nikon A1si confocal laser scanning microscope.

### HE staining

Mouse lung sections (10 μm thick) were stained using an HE stain kit (ScyTek Laboratories, Inc) according to the manufacturer’s instructions. Images were captured at 40× magnification using the virtual slide scanner NanoZoomer 2.0-HT (Hamamatsu Photonics). Fifteen or more images (40×) were obtained to search for micrometastases.

### ECM adhesion assay

ECM adhesion was evaluated using the Chemicon ECM Cell Adhesion Array Kit (MilliporeSigma). Cells were dissociated nonenzymatically with 2 mM EDTA/PBS to prepare a single suspension in serum-free DMEM containing 0.1% BSA. Then, cells were seeded on 96-well plates precoated with various human ECMs and BSA as a negative control. After 1 h of incubation, attached cells were fixed and stained according to the kit procedure and then calorimetrically quantified by absorbance at 570 nm.

### Statistics

Statistical analysis and figure exhibition were performed using GraphPad Prism 9 (GraphPad Software, Inc). All data are expressed as the mean ± standard deviation. Statistical significance between datasets was tested using an unpaired two-sided Student's *t* test. We used one-way ANOVA to compare data from multiple groups. We applied two-way ANOVA to analyze the data affected by two factors. *p* < 0.05 was considered statistically significant.

## Data availability

Some of the data in this study have been published in publicly accessible repositories. Sequence information for PTH-AS is available at the National Center for Biotechnology Information (https://www.ncbi.nlm.nih.gov/) with accession numbers MZ325522, MZ325523, and MZ325524. The raw microarray data analyzed in this article are also available at the Gene Expression Omnibus (https://www.ncbi.nlm.nih.gov/geo/) with accession number GSE176467. Other data used to support the findings of this study are available from the corresponding author upon request.

## Supporting information

This article contains supporting information.

## Conflict of interest

The authors declare that they have no conflicts of interest with the contents of this article.

## Supporting information

Supporting information (revised file).Pdf
