# Supplementary material for: A novel LncRNA PTH-AS upregulates interferon-related DNA damage resistance signature genes and promotes metastasis in human breast cancer xenografts
Source: J Biol Chem. 2022 May 23;298(7):102065. doi: 10.1016/j.jbc.2022.102065 (PMC9198338; doi:10.1016/j.jbc.2022.102065)
Supplement: Supporting information (revised file).Pdf [file mmc1.pdf]

Supporting information

Title:

A Novel LncRNA PTH-AS Upregulates Interferon-Related DNA damage resistance Signature genes and Promotes Metastasis in Human Breast Cancer Xenografts

Authors/Affiliations:

Miho Akimoto*[1], Takao Susa[1], Noriyuki Okudaira[1], Harumi Hisaki[1], Masayoshi Iizuka[2], Hiroko Okinaga[3], Tomoki Okazaki*[1] & Mimi Tamamori-Adachi[1]

[1] Department of Biochemistry, Teikyo University School of Medicine, 2-11-1 Kaga, Itabashi-ku, Tokyo 173-8605, Japan.

[2] Medical Education Center, Teikyo University School of Medicine, 2-11-1 Kaga, Itabashi-ku, Tokyo 173-8605, Japan.

[3] Department of Internal Medicine, Teikyo University School of Medicine, 2-11-1 Kaga, Itabashi-ku, Tokyo 173-8605, Japan.

* Co-corresponding authors

Supplementary Information:

Supplemental Tables S1-S3

Supplemental Figs. S1-S26.

Table S1.
Primers for strand-specific RT (shaded lines) and primer walking

Primer name	Sequence (5' to 3')	Site (NC_000011.10)	
30-R	ATGAAGCCCTCAAGGGAACT	13473890	13473909
29m-R	GATGAATTGGGGGATGTGAT	13477044	13477063
F29m	ACATGCCTGGGATAGAACCT	13477262	13477243
29-R	AGGAAGTTCCAGCCCTCATT	13478161	13478180
F29Long	CTCAGTTTTGTGCTGCTCTAGG	13478305	13478284
28-R	CCTCCTGTCCTAGGGTCCTC	13478731	13478750
F-28	GCTCTTGAGGCTCACCACTC	13478898	13478879
27n-R	GAGCAGCCCCAAGAAACATA	13481639	13481658
F-27n	TATGTTTCTTGGGGCTGCTC	13481658	13481639
27j-R	CACTTTTCCCAGCACCATTT	13484141	13484160
F-27j	GCCATTGCTTTTGGTGTTTT	13484368	13484349
27g2-R	AGCAGAGGCCATGTCTCATC	13486521	13486540
27g-R	GATTGGTCCGTTTTGACAGG	13487101	13487120
RT-4F1	AGGCAATTCTTCCATTGTGG	13487670	13487689
27f-R	TTTCAGTTTGGTTTCCCTGG	13487691	13487710
F-27f	CTTGTGGGGAAAGGCAGATA	13487845	13487826
4-1-F	AAATAAGCAGAGTTTAGTGGCAGAA	13488005	13488029
27e-R	AAATGTCACCTTGACGAGGG	13488065	13488084
4-1-R	CTTTCATTCTTCATCTCTTGGCTTA	13488108	13488084
F-27e	TCCCAGTCACTCCGACTCTT	13488219	13488200
4-2-F	TGATTTAACCTGCGAATTTTCTTTA	13489261	13489285
4-2-R	GAAAATGCCAACTTTCCAAAGAGT	13489372	13489349
RT-4R	TGACGTCAGGTGGTAGCTCA	13490086	13490067
3-F	CAAACTGGAGGTGGAAAT	13490121	13490138
27a2-R	ATTTCCACCTCCAGTTTGGA	13490138	13490119
3-R	TCTTTGGGATCGTTATCCAGA	13490242	13490222
F-27a2	AGAGAAATGGCACGGTGAGT	13490301	13490282
27-R	GATTTGCTGAACCTTAGCGG	13490400	13490419
F-27	AAAACGGATGTCCAGCATTC	13490592	13490573
26e-R	ACACCTCTACCATCCACCCC	13490615	13490596
F-26e	TGCTGAAGGAAACAATCTAGG	13490749	13490729
2-F	CCATTGTATGCCAGTGCCTA	13491622	13491641
2-R	GGACGCAAAATTGCAAGAAA	13491755	13491736
26f-R	AGTCATGTGTTTCACATTTGGA	13491968	13491947
F-26-f	TGCACTTTTAAACCTCTCTTCTACC	13492140	13492116
26g-R	AGAATGGCTGCGTAAGAAGC	13492597	13492578
2-2-F	ATGCATAAGCTGTATTTCACTCACA	13492633	13492657
2-2-R	ATTTTTAATGTTGACTTTATCATTTCG	13492742	13492716
F-26g	CTGTTTTGCCTTGGAATTGG	13492762	13492743
F-26	CTCGTGAAAACCAACCCAAT	13492961	13492942
2-3-F	TGGAGATTTCTTGTGCAAGTT	13493064	13493084
2-3-R	TGCCGCTTAATATCATTGGA	13493229	13493210
25-R	CAGGTGAGTAGTTGCCGCTTA	13493241	13493221
F-25	TTGTGTTTCATTGCCTTGATG	13493434	13493454
1-F	GTAATGCGCCAGTGAAAGGT	13494664	13494683
1-R	CAGTGTGACAGAAGGGAGCA	13494777	13494758
24-R	CACTGAGCATTAAGGAAAAGCA	13495273	13495294
24-F	AAGTTGGATAAGCCCAGTTTGA	13495453	13495432
23-R	GCATTTTACCTTGAAGAAACAACA	13495799	13495822
1-2-F	AACATGGTATTTGTAGAAATGGTACAC	13495819	13495845
1-2-R	CGAAGATCTTGTTCTAAGACATTGTA	13495938	13495913
23-F	TCAGCATCAGCTACTAACATACCTG	13495965	13495941
1-3-F	GCAGACCCCTTAAATGGTG	13495996	13496014
1-3-R	GAGATCCAGAGAATTGGGAGTG	13496096	13496075

Table S2. Primers for qRT-PCR

Primer name	Sequence (5' to 3')	Accession No.	Product size (bp)
huPTH-F	GGAGAGAGTAGAATGGCTGCGT	NM_000315	136
huPTH-R	ATGGCTCTCAACCAAGACATTGTC		
huSTAT1-F	ATGGCAGTCTGGCGGCTGAAT	NM_007315	144
huSTAT1-R	CCAAACCAGGCTGGCACAATTG		
huSTAT2-F	CAGGTCACAGAGTTGCTACAGC	NM_005419	118
huSTAT2-R	CGGTGAACTTGCTGCCAGTCTT		
huIRF9-F	CCACCGAAGTTCCAGGTAACAC	NM_006084	123
huIRF9-R	AGTCTGCTCCAGCAAGTATCGG		
huIFI44-F	GTGAGGTCTGTTTTCCAAGGGC	NM_006417	123
huIFI44-R	CGGCAGGTATTTGCCATCTTTCC		
huOAS1-F	AGGAAAGGTGCTTCCGAGGTAG	NM_016816	127
huOAS1-R	GGACTGAGGAAGACAACCAGGT		
huOAS2-F	GCTTCCGACAATCAACAGCCAAG	NM_016817	158
huOAS2-R	CTTGACGATTTTGTGCCGCTCG		
huIFI27-F	CGTCCTCCATAGCAGCCAAGAT	NM_001130080	147
huIFI27-R	ACCCAATGGAGCCCAGGATGAA		
huIFI6-F	TGATGAGCTGGTCTGCGATCCT	NM_002038	128
huIFI6-R	GTAGCCCATCAGGGCACCAATA		
huIFIT1-F	GCCTTGCTGAAGTGTGGAGGAA	NM_001548	126
huIFIT3-R	GAGCATCTGAGAGTCTGCCCAA		
huMX1-F	GGCTGTTTACCAGACTCCGACA	NM_00114492	143
huMX1-R	CACAAAGCCTGGCAGCTCTCTA		
huISG15-F	CTCTGAGCATCCTGGTGAGGAA	NM_005101	136
huISG15-R	AAGGTCAGCCAGAACAGGTCGT		
huCCL5-F	CCTGCTGCTTTGCCTACATTGC	NM_002985	125
huCCL5-R	ACACACTTGGCGGTTCTTTCGG		
huVEGF-F	CTACCTCCACCATGCCAAGT	NM_001171623	109
huVEGF-R	GCAGTAGCTGCGCTGATAGA		
moVegf-F	TGTACCTCCACCATGCCAAGT	NM_001287056	94
moVegf-R	TGGTAGACATCCATGAACTTG		
huGAPDH-F	GTCTCCTCTGACTTCAACAGCG	NM_002046	131
huGAPDH-R	ACCACCCTGTTGCTGTAGCCAA		
moGAPDH-F	TGCACCACCAACTGCTTAG	NM_001289726	152
moGAPDH-R	GGATGCAGGGATGATGTTC		

Table S2. Primers for qRT-PCR (continued)

Primer name	Sequence (5' to 3')	Accession No.	Product size (bp)
huCDH1-F	GCCTCCTGAAAAGAGAGTGGAAG	NM_004360	131
huCDH1-R	TGGCAGTGTCTCTCCAAATCCG		
huCDH2-F	CCTCCAGAGTTTACTGCCATGAC	NM_001792	149
huCDH2-R	GTAGGATCTCCGCCACTGATTC		
huFN1-F	ACAACACCGAGGTGACTGAGAC	NM_212482	143
huFN1-R	GGACACAACGATGCTTCCTGAG		
huTJP1-F	GTCCAGAATCTCGGAAAAGTGCC	NM_003257	132
huTJP1-R	CTTTCAGCGCACCATACCAACC		
huSNAI1-F	TGCCCTCAAGATGCACATCCGA	NM_005985	133
huSNAI1-R	GGGACAGGAGAAGGGCTTCTC		
huTWIST-F	GCCAGGTACATCGACTTCCTCT	NM_000474	122
huTWIST-R	TCCATCCTCCAGACCGAGAAGG		
huVIM-F	AGGCAAAGCAGGAGTCCACTGA	NM_003380	100
huVIM-R	ATCTGGCGTTCCAGGGACTCAT		
huMMP1-F	ATGAAGCAGCCCAGATGTGGAG	NM_002421	137
huMMP1-R	TGGTCCACATCTGCTCTTGGCA		
huMMP2-F	AGCGAGTGGATGCCGCCTTTAA	NM_004530	138
huMMP2-R	CATTCCAGGCATCTGCGATGAG		
huMMP3-F	CACTCACAGACCTGACTCGGTT	NM_002422	156
huMMP3-R	AAGCAGGATCACAGTTGGCTGG		
huMMP9-F	GCCACTACTGTGCCTTTGAGTC	NM_004994	125
huMMP9-R	CCCTCAGAGAATCGCCAGTACT		
huMMP13-F	CCTTGATGCCATTACCAGTCTCC	NM_002427	97
huMMP13-R	AAACAGCTCCGCATCAACCTGC		
huMMP14-F	CCTTGGACTGTCAGGAATGAGG	NM_004995	146
huMMP14-R	TTCTCCGTGTCCATCCACTGGT		
moIl10-F	CGGGAAGACAATAACTGCACCC	NM_010548	89
moIl10-R	CGGTTAGCAGTATGTTGTCCAGC		
moIl12b-F	TTGAACTGGCGTTGGAAGCACG	NM_001159424	132
moIl12b-R	CCACCTGTGAGTTCTTCAAAGGC		
moCd86-F	ACGTATTGGAAGGAGATTACAGCT	NM_019388	147
moCd86-R	TCTGTCAGCGTTACTATCCCGC		
moCd163-F	GGCTAGACGAAGTCATCTGCAC	NM_001170395	144
moCd163-R	CTTCGTTGGTCAGCCTCAGAGA		
moArg1-F	CATTGGCTTGCGAGACGTAGAC	NM_007482	124
moArg1-R	GCTGAAGGTCTCTTCCATCACC		

Supplementary Table S3.
Antibodies for Western Blotting (WB) and immunofluorescence (IF)

Antibody name	Supplier (Catalog No.)	Clonality (Clone)	Reactivity	Application
Stat1 (42H3) Rabbit mAb	Cell Signaling Technology (#9175)	Rabbit mono (42H3)	Human Monkey	WB (1:1000)
Human Phospho-STAT1 (Y701) Antibody	R&D SYSTEMS (MAB2894)	Rabbit mono (1086B)	Human	WB (1:1000)
Anti-STAT2 Monoclonal Antibody	Rockland (200-301-B65)	Mouse mono (19G8.H2.H6)	Human	WB (1:1000)
Anti-STAT2 pY690 Antibody	Rockland (600-401-A93)	Rabbit poly	Human Mouse	WB (1:1000)
Monoclonal Anti-β-Actin antibody	Sigma-Aldrich (A2228)	Mouse mono (AC-74)	Human Mouse Others	WB (1:3000)
Goat anti-Rabbit IgG (H+L) Cross-Adsorbed Secondary Antibody, HRP	Thermo Fisher Scientific (G-21234)	-	-	WB secondary antibody (1:3000)
Anti-mouse IgG, HRP-linked Antibody	Cell Signaling Technology (#7076)	-	-	WB secondary antibody (1:3000)
Vimentin Polyclonal antibody	Proteintech (28463-1-AP)	Rabbit poly	Human Mouse Rat	IF (1:100)
Purified Rat Anti-Mouse CD31	BD Pharmingen (550274)	Rat mono (MEC 13.3)	Mouse	IF (1:100)
F4/80 Polyclonal antibody	Proteintech (28463-1-AP)	Rabbit poly	Human Mouse	IF (1:250)
Anti-rabbit IgG (H+L), F(ab')2 Fragment (Alexa Fluor® 594 Conjugate)	Cell Signaling Technology (#8889)	-	-	IF secondary antibody (1:400)
Goat Anti-Rat IgG H&L (Alexa Fluor® 594)	abcam (ab150168)	-	-	IF secondary antibody (1:400)

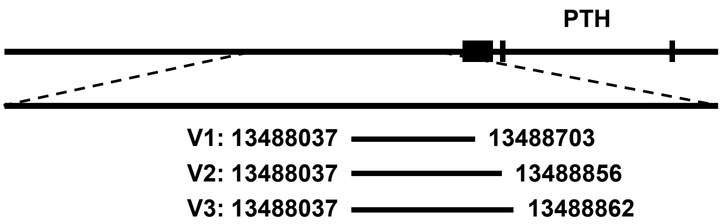

V1

>NC_000011.10:13488037-13488703 Homo sapiens chromosome 11, GRCh38.p13 Primary Assembly
GGAAAGCCTAGACAGACTGAGTGATAGCCCCTCGTCAAGGTGACATTTAAGCCAAGAGATGAAGAATGAA
AGAAGGGGAGGCCACATGAAAAAGGCAAGCCCTGGAATAGTGAGAACAGAAGCTTTGGAGGTGATGAGCC
CGGTGTGTTCTGGGAATTGATGAAAGAGTCGGAGTGACTGGGACCCGTAGGGGTGGGAGGTTCAGGAAAT
GAGTGTGGAGAAAGAGACACAAGGCCTTCAAAGCAGATTAATAAGGGGTGTGGCTTCCACCTAGACTTCA
GTGACACTGCTAAATGGCAGAGCTGTTCTAAGTGTTTTATCTGTATGAACTGTGCTTCAGTGAGTTTTGG
TTGTAAGCCCTGTTTTACAGAGATGGCAATTAATTAATTAAGACCCAACGTACTGAATCAGCTTGCCTCA
GGGTCCCTTGGCTGAGCCAGGATCACTCCTCTATGTTGTCTTTCAAGATATGTAGAAAGAAATCCTTTAG
AAAGGCAAATGTCGATTTCTAATGCAGACAAAACATAACTAACTGCACTAAGTGGTCATCATAGGCTCAG
TTGTCTCAATTTTGTGGTACTGGGTCAGTTTCTCTGTTTTAGGTTTGGATTCCCTGCATACAGACACTTT
AGAAGTGATGGCACCTCTCCCAACATCCCCATGCCCC

V2

>NC_000011.10:13488037-13488856 Homo sapiens chromosome 11, GRCh38.p13 Primary Assembly
GGAAAGCCTAGACAGACTGAGTGATAGCCCCTCGTCAAGGTGACATTTAAGCCAAGAGATGAAGAATGAA
AGAAGGGGAGGCCACATGAAAAAGGCAAGCCCTGGAATAGTGAGAACAGAAGCTTTGGAGGTGATGAGCC
CGGTGTGTTCTGGGAATTGATGAAAGAGTCGGAGTGACTGGGACCCGTAGGGGTGGGAGGTTCAGGAAAT
GAGTGTGGAGAAAGAGACACAAGGCCTTCAAAGCAGATTAATAAGGGGTGTGGCTTCCACCTAGACTTCA
GTGACACTGCTAAATGGCAGAGCTGTTCTAAGTGTTTTATCTGTATGAACTGTGCTTCAGTGAGTTTTGG
TTGTAAGCCCTGTTTTACAGAGATGGCAATTAATTAATTAAGACCCAACGTACTGAATCAGCTTGCCTCA
GGGTCCCTTGGCTGAGCCAGGATCACTCCTCTATGTTGTCTTTCAAGATATGTAGAAAGAAATCCTTTAG
AAAGGCAAATGTCGATTTCTAATGCAGACAAAACATAACTAACTGCACTAAGTGGTCATCATAGGCTCAG
TTGTCTCAATTTTGTGGTACTGGGTCAGTTTCTCTGTTTTAGGTTTGGATTCCCTGCATACAGACACTTT
AGAAGTGATGGCACCTCTCCCAACATCCCCATGCCCCACTTCTGTCTTACCTGGAGTACCTAAGCTTGTT
TTTCCTACAGTCAATGGACAGGCAAAGTTTGAAACCAAAGTTACTATGTTGGATAATGGGCTTTGTGTGG
CATCTTAAAATAAGTTTGGAAGCTTTTATATGGCAGGAAGTAAATACTTT

V3

>NC_000011.10:13488037-13488862 Homo sapiens chromosome 11, GRCh38.p13 Primary Assembly
GGAAAGCCTAGACAGACTGAGTGATAGCCCCTCGTCAAGGTGACATTTAAGCCAAGAGATGAAGAATGAA
AGAAGGGGAGGCCACATGAAAAAGGCAAGCCCTGGAATAGTGAGAACAGAAGCTTTGGAGGTGATGAGCC
CGGTGTGTTCTGGGAATTGATGAAAGAGTCGGAGTGACTGGGACCCGTAGGGGTGGGAGGTTCAGGAAAT
GAGTGTGGAGAAAGAGACACAAGGCCTTCAAAGCAGATTAATAAGGGGTGTGGCTTCCACCTAGACTTCA
GTGACACTGCTAAATGGCAGAGCTGTTCTAAGTGTTTTATCTGTATGAACTGTGCTTCAGTGAGTTTTGG
TTGTAAGCCCTGTTTTACAGAGATGGCAATTAATTAATTAAGACCCAACGTACTGAATCAGCTTGCCTCA
GGGTCCCTTGGCTGAGCCAGGATCACTCCTCTATGTTGTCTTTCAAGATATGTAGAAAGAAATCCTTTAG
AAAGGCAAATGTCGATTTCTAATGCAGACAAAACATAACTAACTGCACTAAGTGGTCATCATAGGCTCAG
TTGTCTCAATTTTGTGGTACTGGGTCAGTTTCTCTGTTTTAGGTTTGGATTCCCTGCATACAGACACTTT
AGAAGTGATGGCACCTCTCCCAACATCCCCATGCCCCACTTCTGTCTTACCTGGAGTACCTAAGCTTGTT
TTTCCTACAGTCAATGGACAGGCAAAGTTTGAAACCAAAGTTACTATGTTGGATAATGGGCTTTGTGTGG
CATCTTAAAATAAGTTTGGAAGCTTTTATATGGCAGGAAGTAAATACTTTTGCTAA

Figure S1 Sequence of transcript of PTH-AS. The sequences of the three transcripts of PTH-AS (V1, V2 and V3 in Fig. 1*C*) determined by 5′ and 3′-RACE are shown. These sequence information can also be found at the National Center for Biotechnology Information (NCBI, https: //www.ncbi.nlm.nih.gov/) with accession numbers MZ325522 (V1), MZ325523 (V2), and MZ325524 (V3).

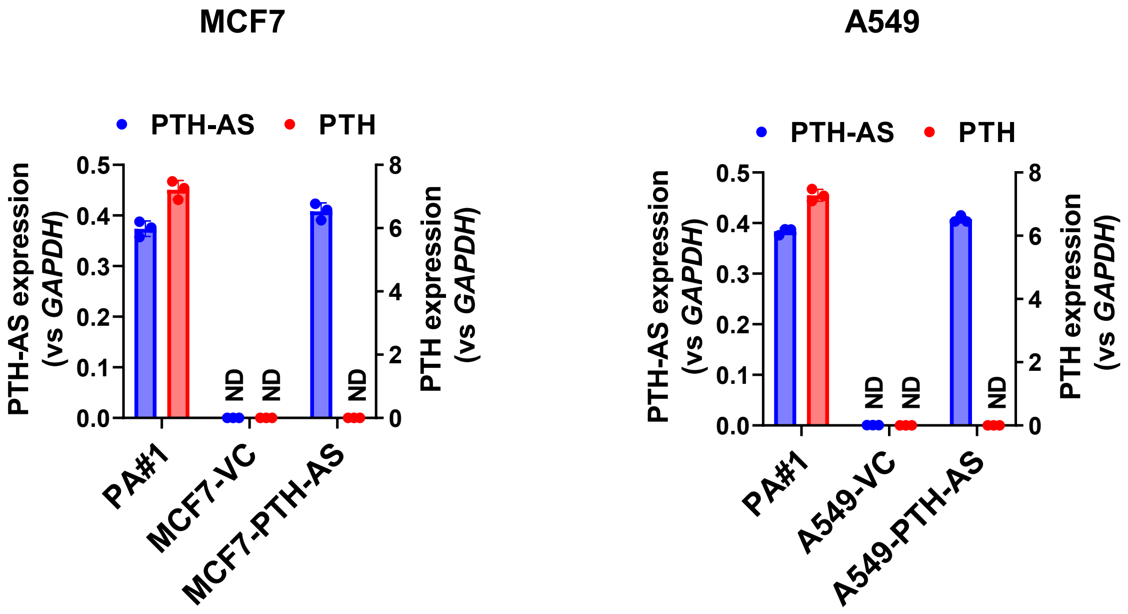

Figure S2 PTH expression in cancer cells overexpressing PTH-AS. PTH-AS was transiently overexpressed in human lung cancer A549 and human breast cancer MCF-7 cells, PTH mRNA levels were quantified by qRT-PCR and normalized to *GAPDH* (n = 3). All data are shown as the mean ± SD. ND, not determined.

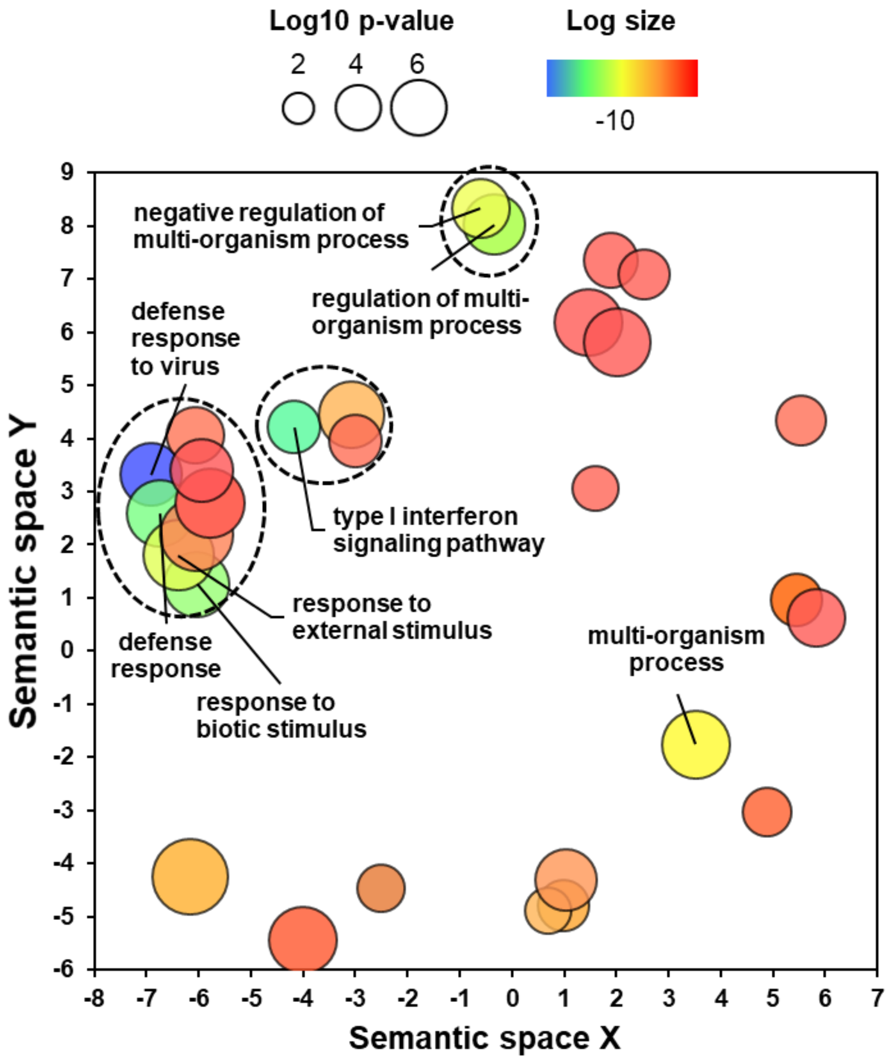

Figure S3 Gene Ontology-based semantic similarity. Semantic similarity based on GO-BP data. The size and color of the circles indicate the log 10 p-value and log size for each GO, respectively. Dotted lines indicate immune response-related clusters .

A

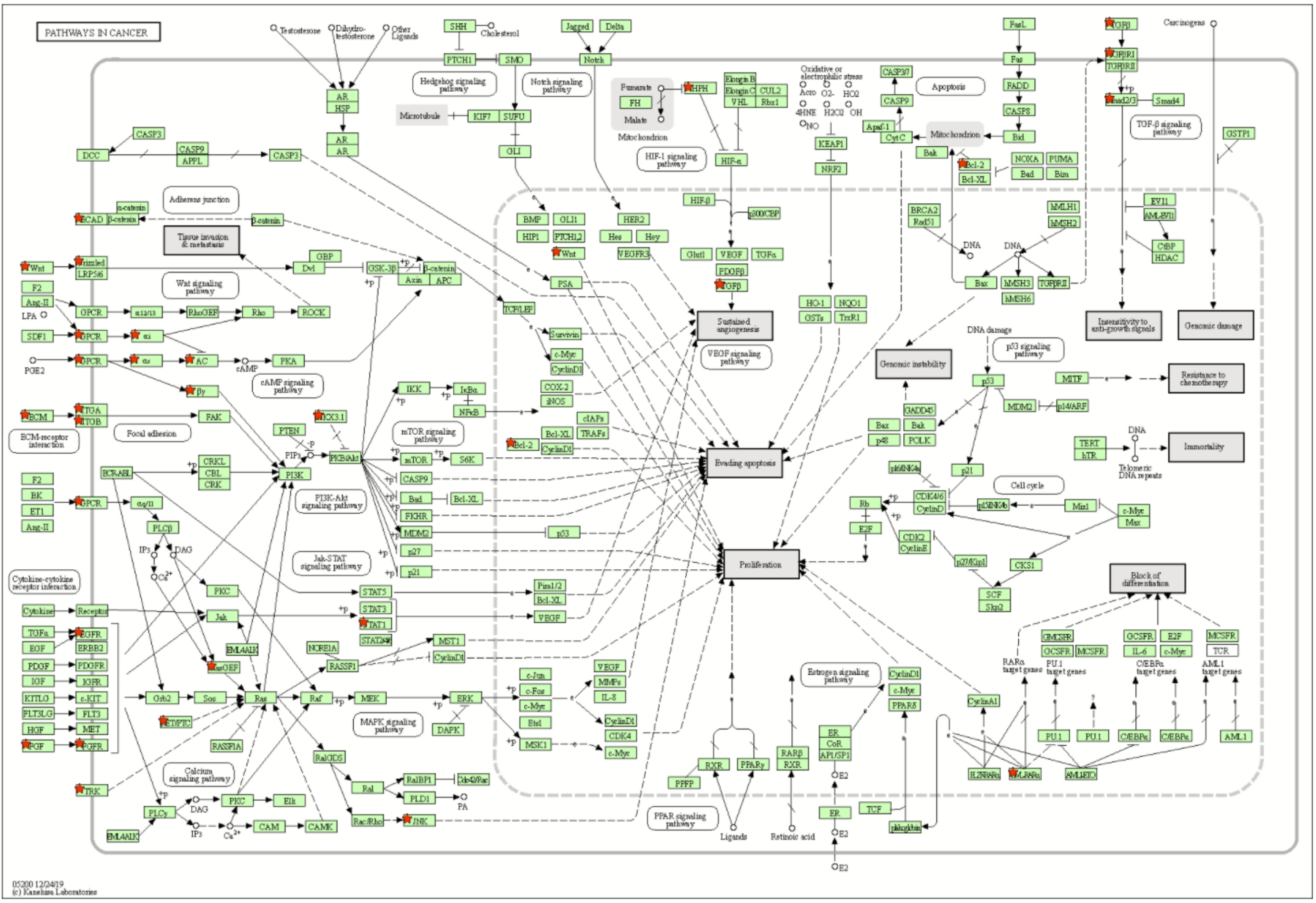
PATHWAYS IN CANCER
Hedgehog signaling pathway
Notch signaling pathway
Apoptosis
HIF-1 signaling pathway
TGF-β signaling pathway
Adherens junction
Tissue invasion & metastasis
Wnt signaling pathway
Sustained angiogenesis
Insensitivity to anti-growth signals
Genomic damage
Genomic instability
Resistance to chemotherapy
Evading apoptosis
Immortality
Proliferation
Block of differentiation
Focal adhesion
ECM-receptor interaction
mTOR signaling pathway
PI3K-Akt signaling pathway
Jak-STAT signaling pathway
Cytokine-cytokine receptor interaction
MAPK signaling pathway
Calcium signaling pathway
VEGF signaling pathway
p53 signaling pathway
Cell cycle
Estrogen signaling pathway
PPAR signaling pathway
05200 12/24/19
(c) Kanehisa Laboratories

B

GENE SYMBOL	GENE NAME	PA6 vs VC1
BCL2	BCL2, apoptosis regulator(BCL2)	4.64148
CXCR4	C-X-C motif chemokine receptor 4(CXCR4)	1.80124
GNAI1	G protein subunit alpha i1(GNAI1)	3.43649
GNG4	G protein subunit gamma 4(GNG4)	NULL
GNAS	GNAS complex locus(GNAS)	0.54919
NKX3-1	NK3 homeobox 1(NKX3-1)	1.39623
RASGRP1	RAS guanyl releasing protein 1(RASGRP1)	0.29108
SMAD3	SMAD family member 3(SMAD3)	1.46143
WNT3A	Wnt family member 3A(WNT3A)	2.75035
WNT6	Wnt family member 6(WNT6)	1.82153
ADCY1	adenylate cyclase 1(ADCY1)	6.15342
BDKRB1	bradykinin receptor B1(BDKRB1)	2.94848
BDKRB2	bradykinin receptor B2(BDKRB2)	2.24512
EGLN3	egl-9 family hypoxia inducible factor 3(EGLN3)	1.72352
EGFR	epidermal growth factor receptor(EGFR)	NULL
FGF13	fibroblast growth factor 13(FGF13)	NULL
FGF22	fibroblast growth factor 22(FGF22)	NULL
FGFR3	fibroblast growth factor receptor 3(FGFR3)	0.75282
FZD8	frizzled class receptor 8(FZD8)	NULL
ITGA2	integrin subunit alpha 2(ITGA2)	NULL
LAMB3	laminin subunit beta 3(LAMB3)	4.88485
LAMC1	laminin subunit gamma 1(LAMC1)	0.98722
MAPK9	mitogen-activated protein kinase 9(MAPK9)	0.40462
PML	promyelocytic leukemia(PML)	2.33254
PTGER2	prostaglandin E receptor 2(PTGER2)	11.62813
PTGER4	prostaglandin E receptor 4(PTGER4)	0.18773
RET	ret proto-oncogene(RET)	1.17033
STAT1	signal transducer and activator of transcription 1(STAT1)	11.80227
TGFB1	transforming growth factor beta 1(TGFB1)	1.59476
TGFB2	transforming growth factor beta 2(TGFB2)	NULL
TGFBR1	transforming growth factor beta receptor 1(TGFBR1)	0.54694
TPM3	tropomyosin 3(TPM3)	0.31519

Figure S4 'Pathways in cancer'-relayed genes altered in T47D cells with PTH-AS expression. A. KEGG pathway for 'pathways in cancer' (hsa05200). Red stars indicate genes whose expression was affected by PTH expression in T47D cells. **B.** A list of 'pathways in cancer'-related genes whose expression was upregulated (>2.0; pink) or downregulated (<0.6; blue) in T47D-PA6 cells compared with T47D-VC1 cells. The expression level of each gene is shown as a relative value with the value of T47D-VC1 as 1 based on the data in the microarray analysis.

A

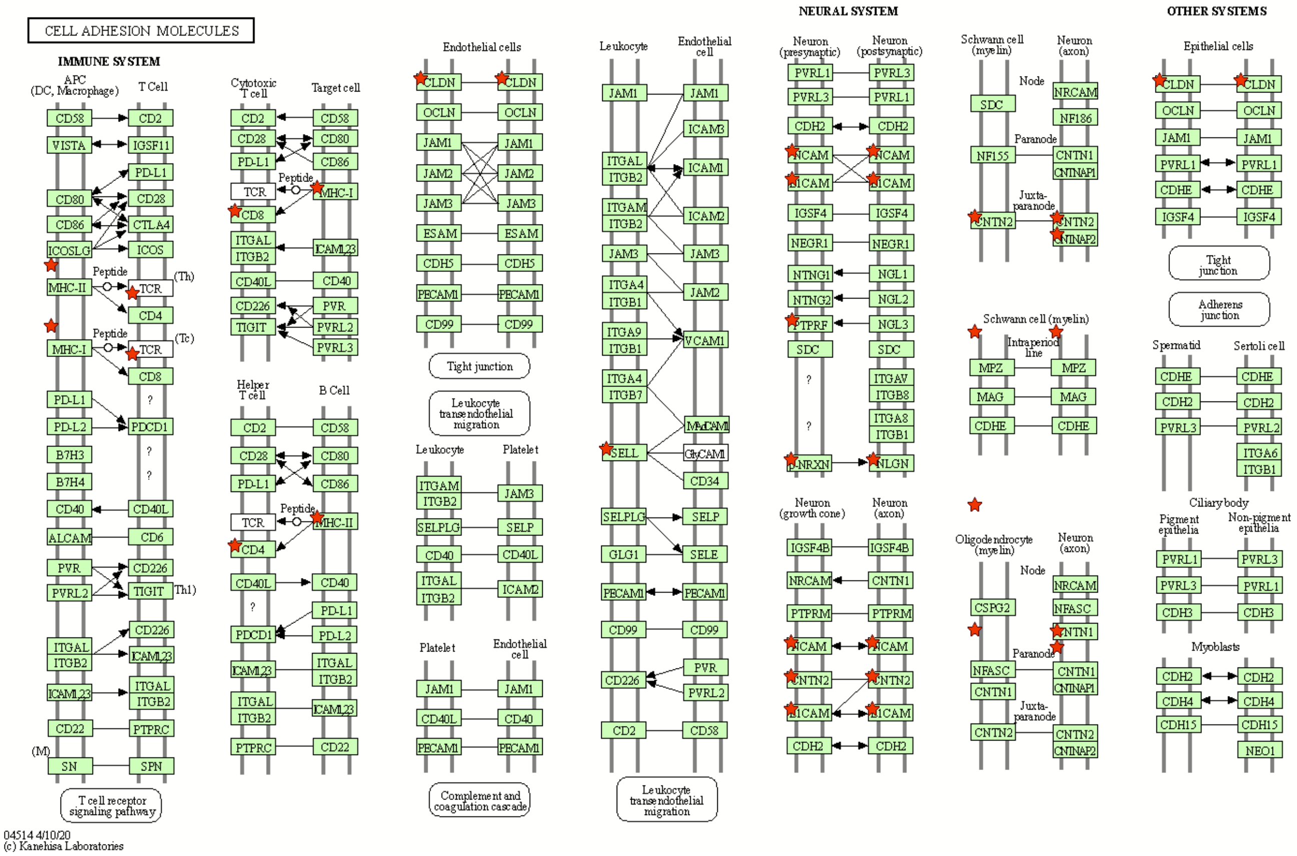

B

GENE SYMBOL	GENE NAME	PA6 vs VC1
CLDN1	claudin 1(CLDN1)	0.742097
CLDN16	claudin 16(CLDN16)	NULL
CNTNAP2	contactin associated protein-like 2(CNTNAP2)	0.213782
HLA-A	major histocompatibility complex, class I, A(HLA-A)	9.182066
HLA-B	major histocompatibility complex, class I, B(HLA-B)	4.23503
HLA-E	major histocompatibility complex, class I, E(HLA-E)	3.165355
HLA-F	major histocompatibility complex, class I, F(HLA-F)	4.310169
HLA-G	major histocompatibility complex, class I, G(HLA-G)	3.425848
HLA-DQA1	major histocompatibility complex, class II, DQ alpha 1(HLA-DQA1)	NULL
HLA-DQA2	major histocompatibility complex, class II, DQ alpha 2(HLA-DQA2)	0.655302
MPZL1	myelin protein zero like 1(MPZL1)	0.618878
NCAM2	neural cell adhesion molecule 2(NCAM2)	0.376765
NLGN4X	neuroligin 4, X-linked(NLGN4X)	18.39267
VCAN	versican(VCAN)	0.137314

Figure S5 'Cell adhesion molecules'-related genes altered in T47D cells with PTH-AS expression. A. KEGG pathway for 'cell adhesion molecules' (hsa04514). Red stars indicate genes whose expression altered with PTH expression in T47D cells. **B.** A list of 'Cell adhesion molecules'-related genes whose expression was upregulated (>2.0; pink) or downregulated (<0.6; blue) in T47D-PA6 cells compared with T47D-VC1 cells. The expression level of each gene is shown as a relative value with the value of T47D-VC1 as 1 based on the data in the microarray analysis.

A

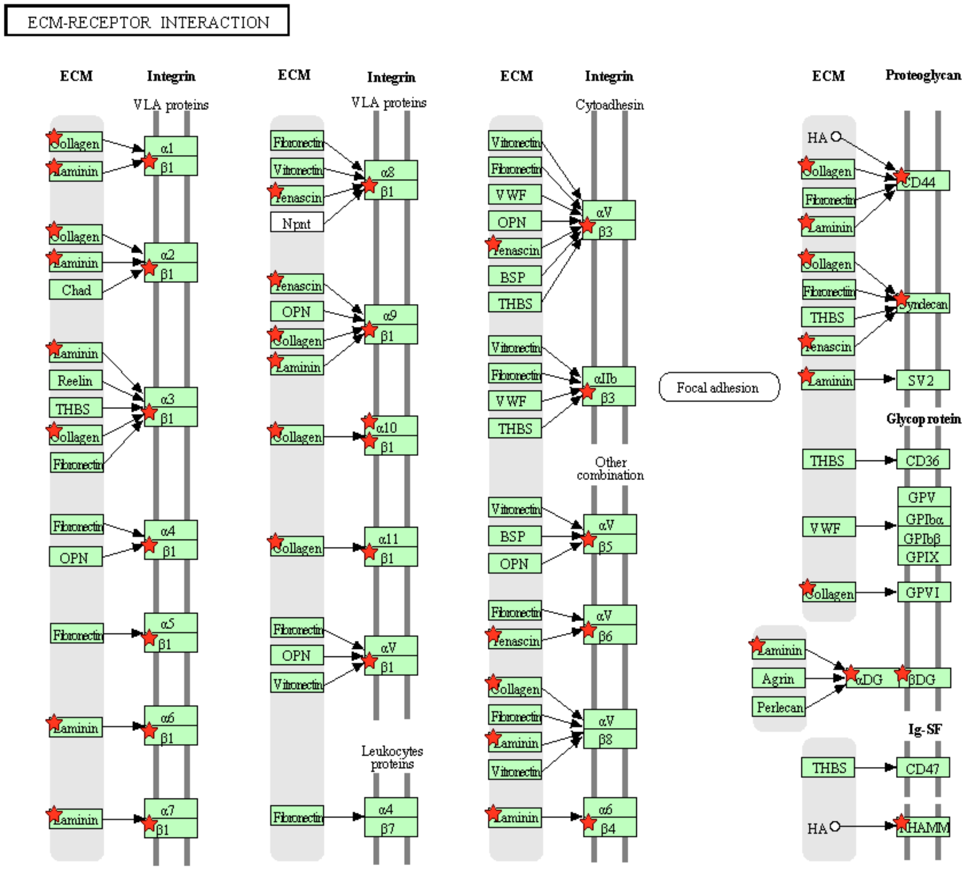

B

GENE SYMBOL	GENE NAME	PA6 vs VC1
ATP2B4	ATPase plasma membrane Ca2+ transporting 4(ATP2B4)	1.68505
GNAS	GNAS complex locus(GNAS)	0.42786
ADORA2A	adenosine A2a receptor(ADORA2A)	1.29563
ADCY1	adenylate cyclase 1(ADCY1)	10.0563
ADCY4	adenylate cyclase 4(ADCY4)	3.39188
ADRA1B	adrenoceptor alpha 1B(ADRA1B)	3.71824
ADRB1	adrenoceptor beta 1(ADRB1)	2.36633
BDKRB1	bradykinin receptor B1(BDKRB1)	7.57073
BDKRB2	bradykinin receptor B2(BDKRB2)	4.06732
CACNA1F	calcium voltage-gated channel subunit alpha1 F(CACNA1F)	2.33213
CAMK2B	calcium/calmodulin dependent protein kinase II beta(CAMK2B)	1.03793
CHRM1	cholinergic receptor muscarinic 1(CHRM1)	0.75289
CHRM2	cholinergic receptor muscarinic 2(CHRM2)	1.57919
CHRM3	cholinergic receptor muscarinic 3(CHRM3)	1.38226
ITPKA	inositol-trisphosphate 3-kinase A(ITPKA)	0.73166
PLCB2	phospholipase C beta 2(PLCB2)	1.11905
PLCE1	phospholipase C epsilon 1(PLCE1)	2.2903
PTAFR	platelet activating factor receptor(PTAFR)	4.34246
PTGER3	prostaglandin E receptor 3(PTGER3)	1.14028
P2RX5	purinergic receptor P2X 5(P2RX5)	3.81303

Figure S6 'ECM-receptor interaction'–related genes altered in T47D cells with PTH-AS expression. A. KEGG pathway for 'ECM-receptor interaction' (hsa04512). Red stars indicate genes whose expression is modified with PTH expression in T47D cells. **B.** A list of 'JAK-STAT signalling pathway'-related genes whose expression was upregulated (>2.0; pink) or downregulated (<0.6; blue) in T47D-PA6 cells compared with T47D-VC1 cells. The expression level of each gene is shown as a relative value with the value of T47D-VC1 as 1 based on the data in the microarray analysis.

A

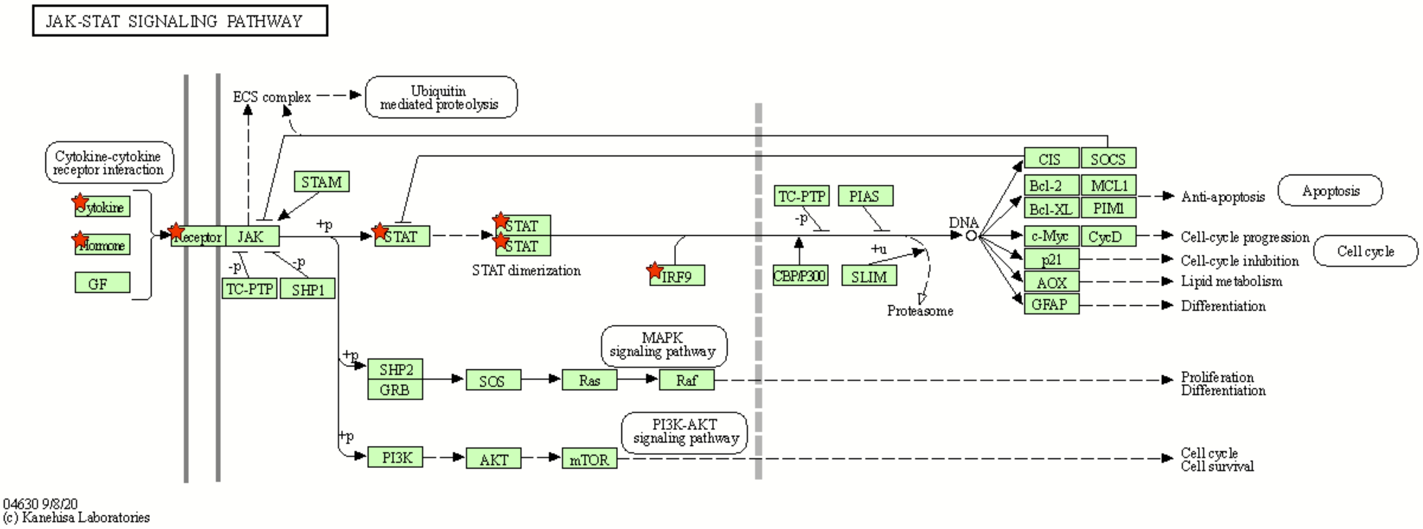

B

GENE SYMBOL	GENE NAME	PA6 vs VC1
CNTFR	ciliary neurotrophic factor receptor(CNTFR)	1.084043
GHR	growth hormone receptor(GHR)	0.434186
IFNB1	interferon beta 1(IFNB1)	4.279754
IFNL1	interferon lambda 1(IFNL1)	16.6653
IFNL2	interferon lambda 2(IFNL2)	6.886468
IFNL3	interferon lambda 3(IFNL3)	7.200455
IRF9	interferon regulatory factor 9(IRF9)	8.92854
IL20	interleukin 20(IL20)	0.248452
IL6R	interleukin 6 receptor(IL6R)	0.370111
IL9R	interleukin 9 receptor(IL9R)	0.297033
OSMR	oncostatin M receptor(OSMR)	1.705304
STAT1	signal transducer and activator of transcription 1(STAT1)	11.51095
THPO	thrombopoietin(THPO)	2.413087

Figure S7 'JAK-STAT signaling pathway'-related genes altered in T47D cells with PTH-AS expression. A. KEGG pathway for 'JAK-STAT signaling pathway' (hsa04630). Red stars indicate genes whose expression alters with PTH expression in T47D cells. **B.** A list of 'JAK-STAT signaling pathway'-related genes whose expression was upregulated (>2.0; pink) or downregulated (<0.6; blue) in T47D-PA6 cells compared with T47D-VC1 cells. The expression level of each gene is shown as a relative value with the value of T47D-VC1 as 1 based on the data in the microarray analysis.

A

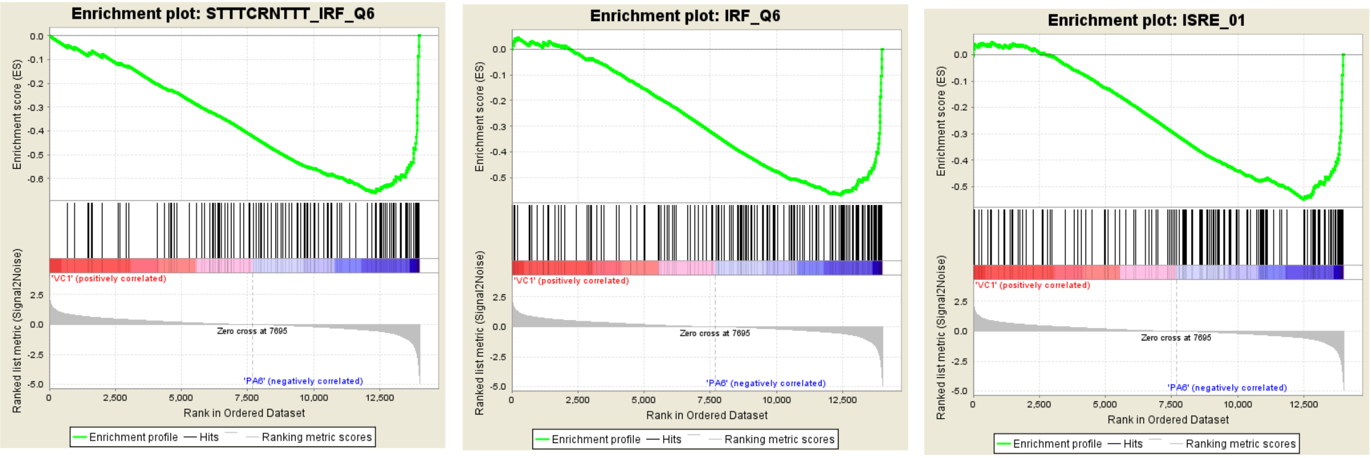

B

Matrix ID	Matrix Name	*P*-value (< 0.001)
MA0517.1	STAT1::STAT2	1.29E-12
MA1418.1	IRF3	3.37E-10
MA0528.1	ZNF263	7.07E-10
MA0051.1	IRF2	2.11E-09
MA0155.1	INSM1	5.68E-07
MA0039.3	KLF4	2.11E-06
MA0050.2	IRF1	2.86E-06
MA0652.1	IRF8	3.67E-06
MA0872.1	TFAP2A(var.3)	2.02E-05
MA0513.1	SMAD2::SMAD3::SMAD4	3.41E-05
MA0073.1	RREB1	3.43E-05
MA0753.1	ZNF740	5.25E-05
MA0815.1	TFAP2C(var.3)	6.59E-05
MA0653.1	IRF9	6.93E-05
MA1102.1	CTCFL	8.12E-05
MA0813.1	TFAP2B(var.3)	0.000123
MA1419.1	IRF4	0.000124
MA1107.1	KLF9	0.000184
MA1114.1	PBX3	0.000199
MA0597.1	THAP1	0.000212
MA0516.1	SP2	0.000246
MA0116.1	Znf423	0.000265
MA0746.1	SP3	0.000317
MA0733.1	EGR4	0.000421
MA0137.3	STAT1	0.000482
MA0471.1	E2F6	0.00055
MA0696.1	ZIC1	0.000582
MA0751.1	ZIC4	0.000613

Figure S8 Expression of STAT1 downstream gene in PTH-AS expressing T47D cells. *A*. Gene set enrichment analysis (GSEA). The relationship between PTH-AS expression and the expression of genes regulated by transcription factor related to JAK-STAT signalling in T47D cells is shown. *B*. Analysis by PScan. The list shows candidate transcription factors that are predicted to bind to the promoter region of genes whose expression was significantly affected by PTH-AS expression in T47D cells.

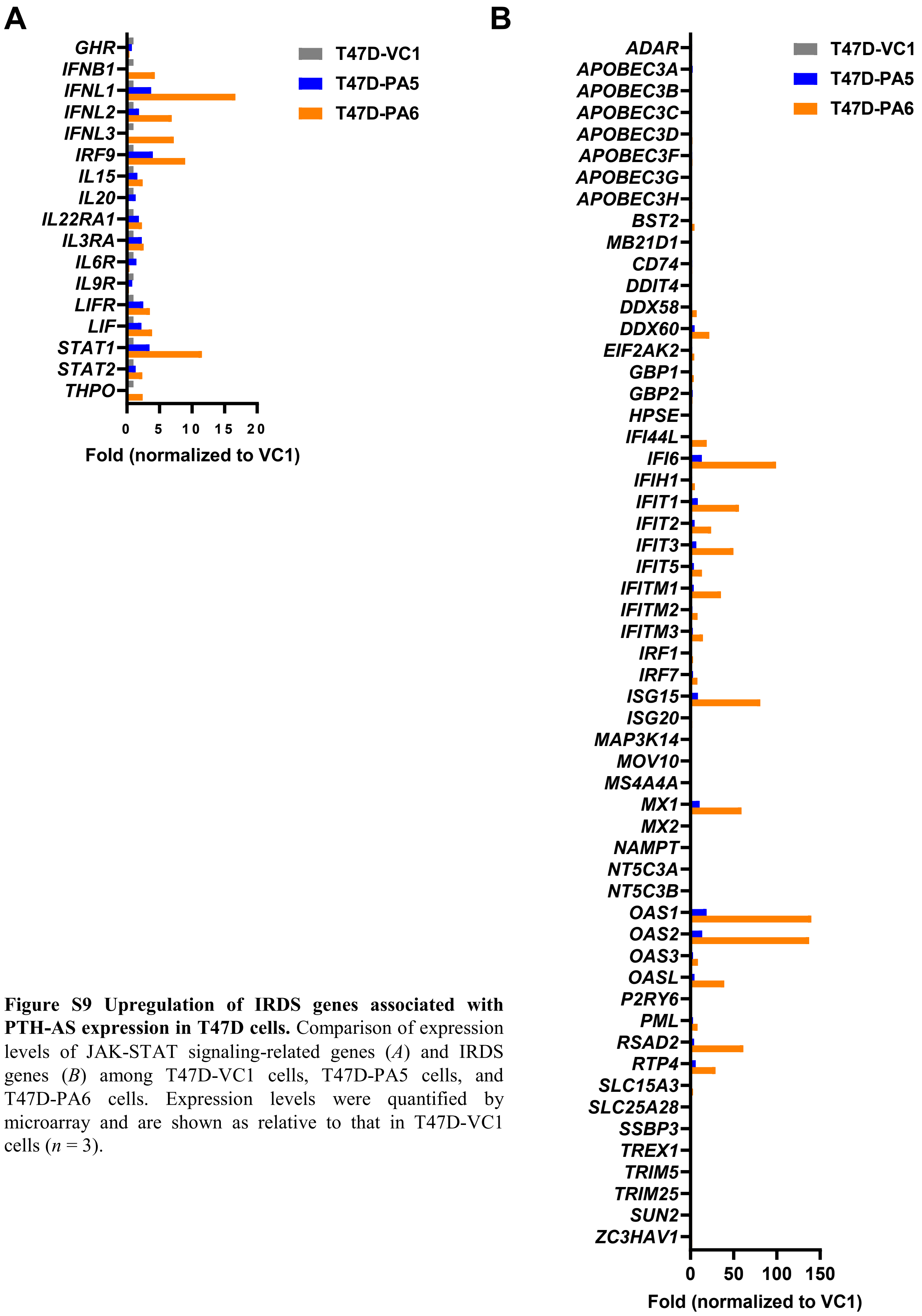

Figure S9 Upregulation of IRDS genes associated with PTH-AS expression in T47D cells. Comparison of expression levels of JAK-STAT signaling-related genes (*A*) and IRDS genes (*B*) among T47D-VC1 cells, T47D-PA5 cells, and T47D-PA6 cells. Expression levels were quantified by microarray and are shown as relative to that in T47D-VC1 cells (n = 3).

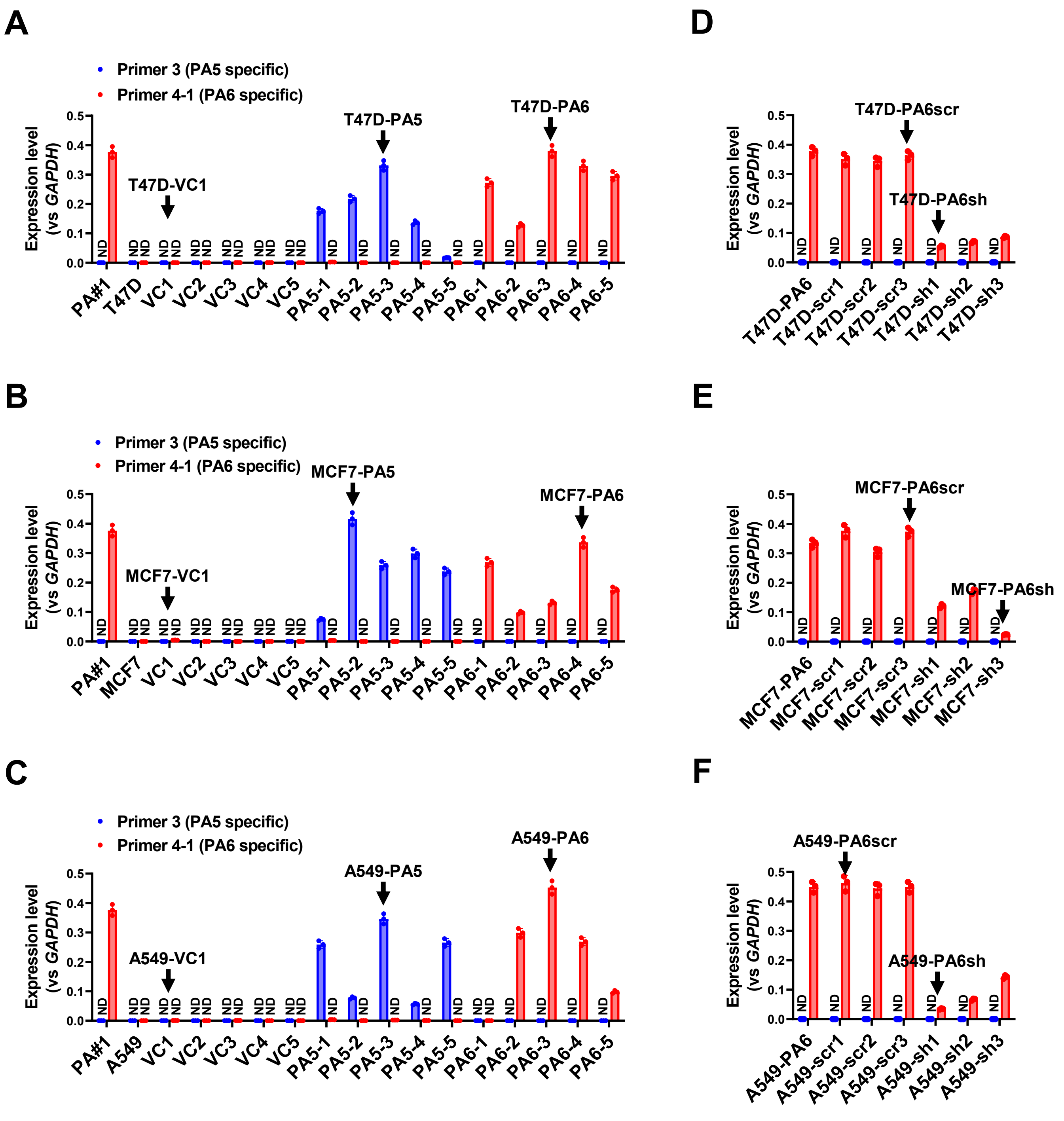

Figure S10 Stable expression of PTH-AS and its shRNA-mediated knockdown. *A-C*, Plasmid vectors containing full-length PTH-AS (PA6) or incomplete PTH-AS (PA5) were transfected into T47D (*A*), MCF-7 (*B*), and A549 (*C*) cells to establish stable cell lines after G418 selection. Finally, five cells with high expression levels of the gene of interest were selected and one of them was used in subsequent experiments. *D-F*, PTH-AS-specific shRNA was introduced into each cell expressing full-length PTH-AS (PA6) prepared in A-C, and the expression of PTH-AS was suppressed. Of the three shRNAs (see experimental procedures), # 1 with the highest knockdown efficiency was used. Of the three clones finally selected, the one with the most effective suppression of PTH-AS expression was used in subsequent experiments. The cells used in this experiment, including cells into which vector control or scrambled shRNA have been introduced, are indicated by arrows. Expression of PA5 and PA6 was confirmed by qRT-PCR using primer sets specific to each. All data are shown as the mean ± SD. ND, not determined.

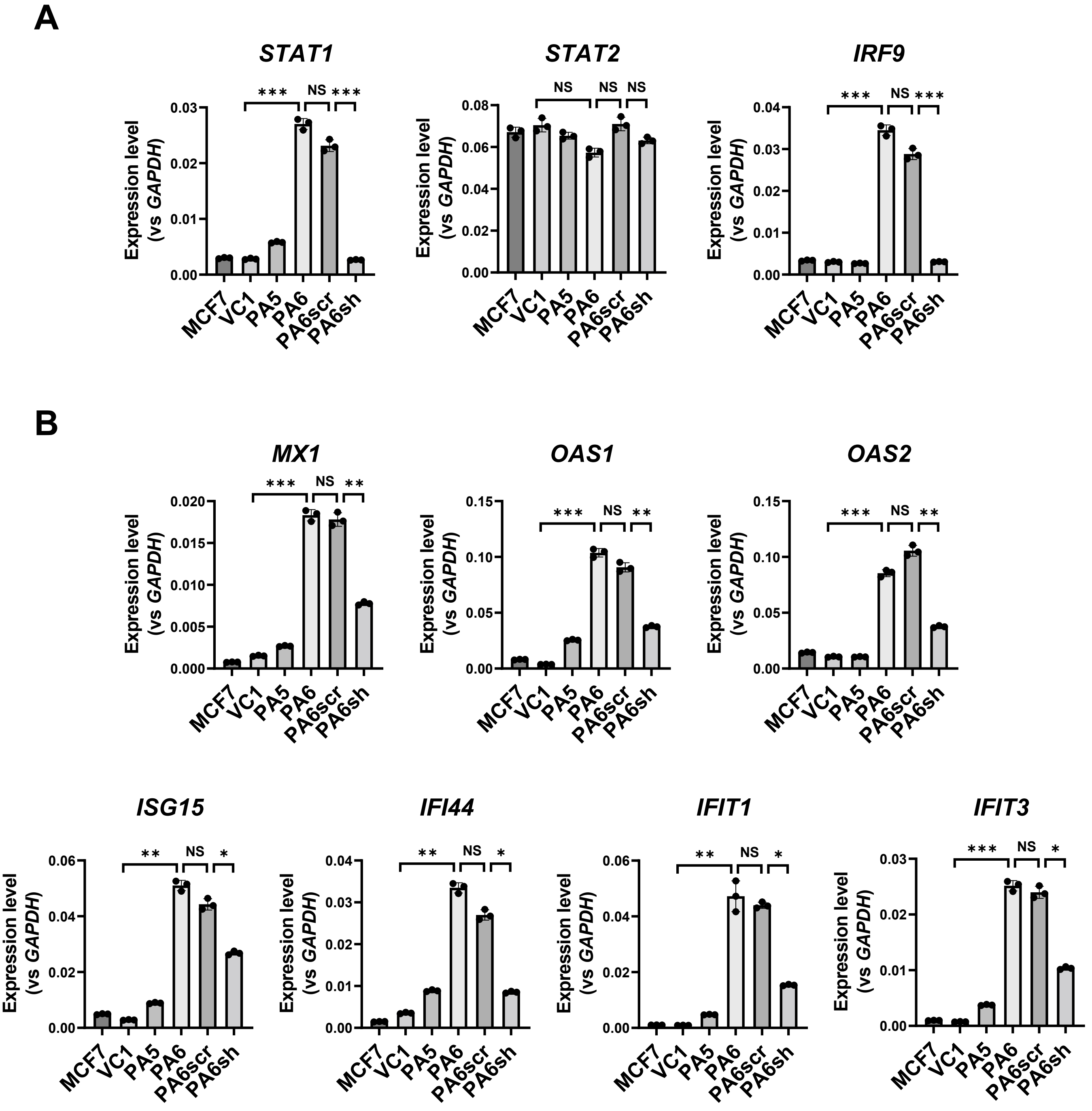

Figure S11 Upregulation of the STAT1 target IRDS gene associated with PTH-AS expression in MCF7 cells. Expression levels of JAK-STAT signaling-related genes (*A*) and IRDS genes (*B*) in MCF7 cells with different PTH-AS expression levels. The mRNA level of each gene was quantified by qRT-PCR and normalized by *GAPDH* expression (n = 3). All data are shown as the mean ± SD. NS, not significant. **P* < 0.05, ***P* < 0.01, ****P* < 0.001 by one-way ANOVA.

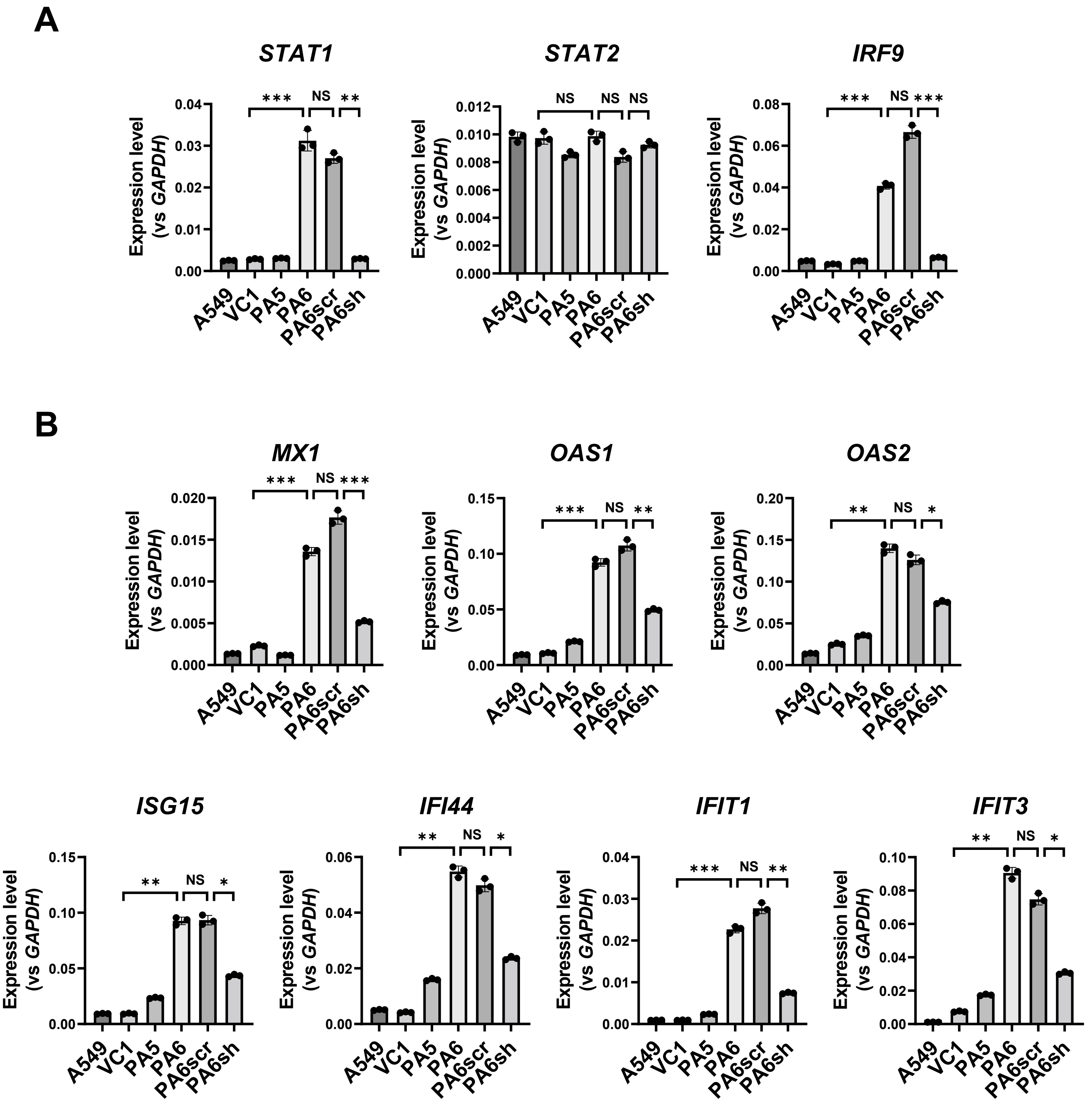

Figure S12 Upregulation of the STAT1 target IRDS gene associated with PTH-AS expression in A549 cells. Expression levels of JAK-STAT signaling-related genes (*A*) and IRDS genes (*B*) in A549 cells with different PTH-AS expression levels. The mRNA level of each gene was quantified by qRT-PCR and normalized by *GAPDH* expression (n = 3). All data are shown as the mean ± SD. NS, not significant. *$P < 0.05$, **$P < 0.01$, ***$P < 0.001$ by one-way ANOVA.

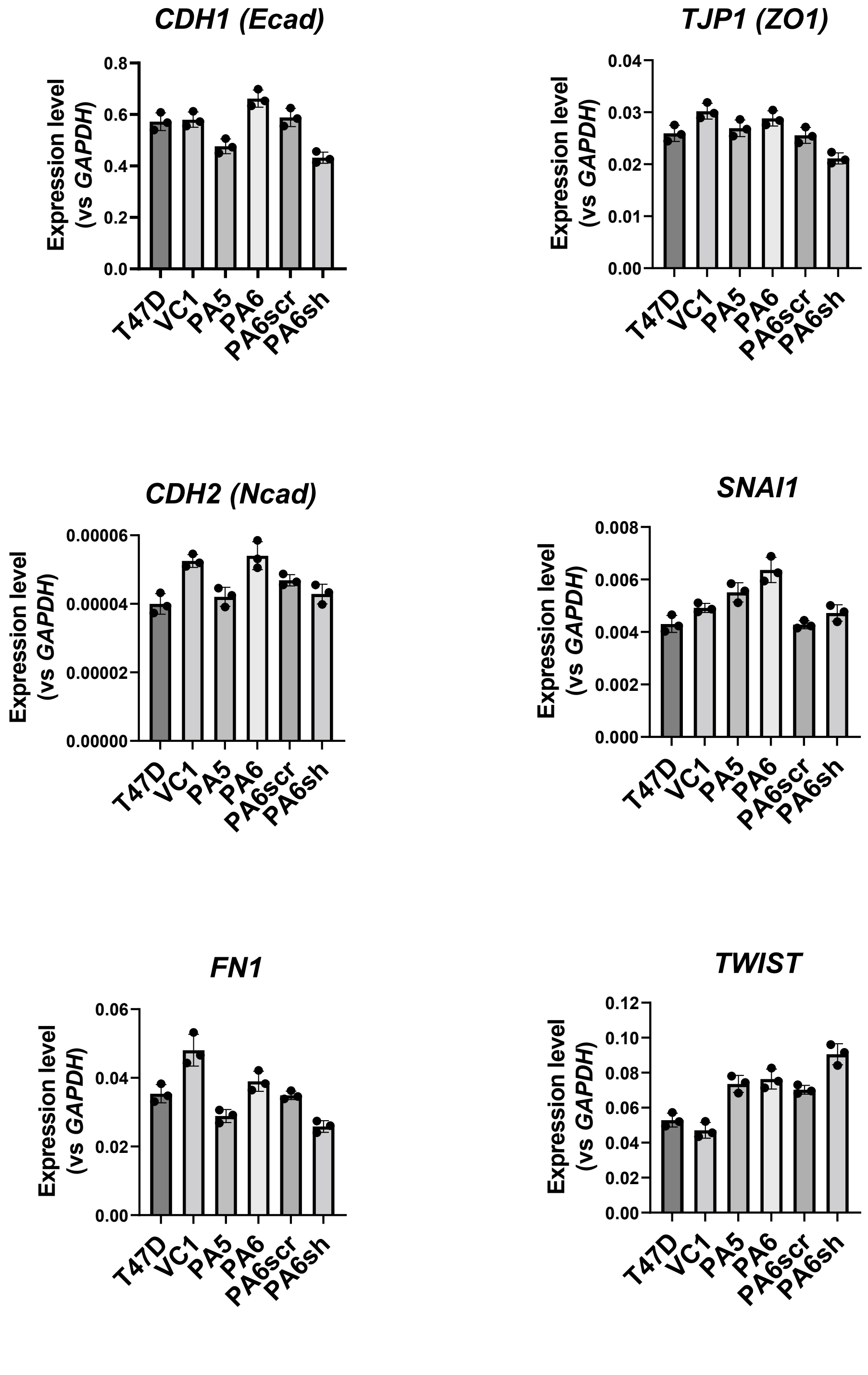

Figure S13 EMT marker expression in T47D cells expressing PTH-AS. The gene expression levels of epithelial markers (*CDH1* and *TJP1*) and mesenchymal markers (*CDH2*, SNAI1, *SNAI1*, *FN*, *TWIST*) were quantified by qRT-PCR and normalized to *GAPDH* ($n = 3$). All data are shown as the mean ± SD.

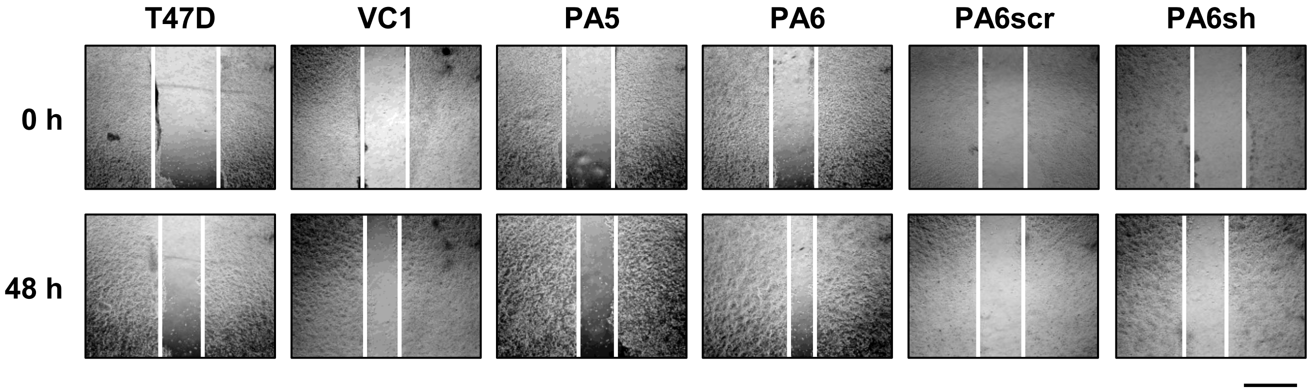

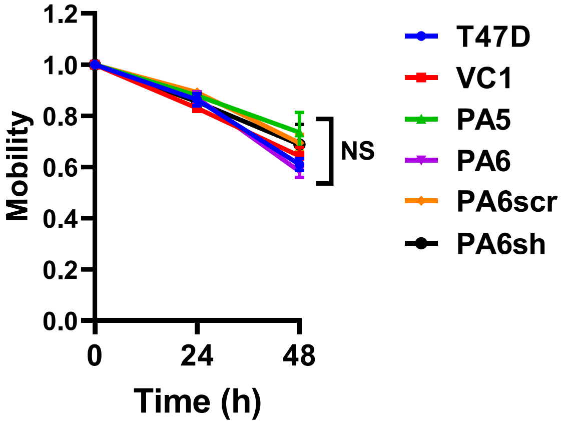

Figure S14 Effect of PTH-AS expression on 2D of T47D cells. Wound healing assay. Artificial wounds are formed by scratching the cell monolayer prepared on a 12-well plate, and images are captured at 0, 24, and 48 h (40×). Scale bar, 500 µm. The width of the gap of the wounds at each time is measured using ImageJ software, and the motility is represented with the value at 0 h as 1 (n = 3). The white line indicates the edge of the gap. All data are shown as the mean ± SD. NS, not significant by one-way ANOVA.

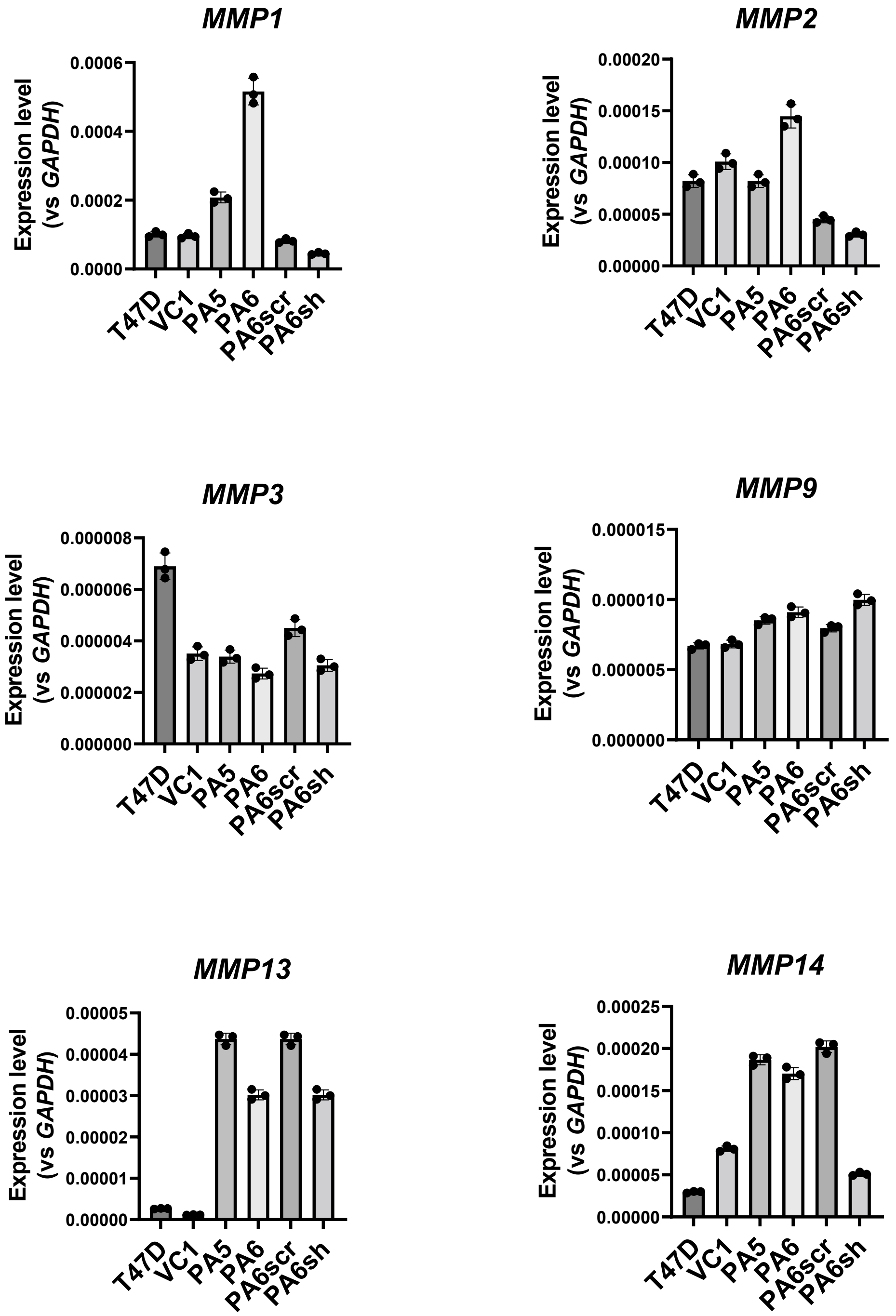

Figure S15 MMP expression in T47D cells expressing PTH-AS. mRNA levels were quantified by qRT-PCR and normalized to *GAPDH* ($n = 3$). All data are shown as the mean ± SD.

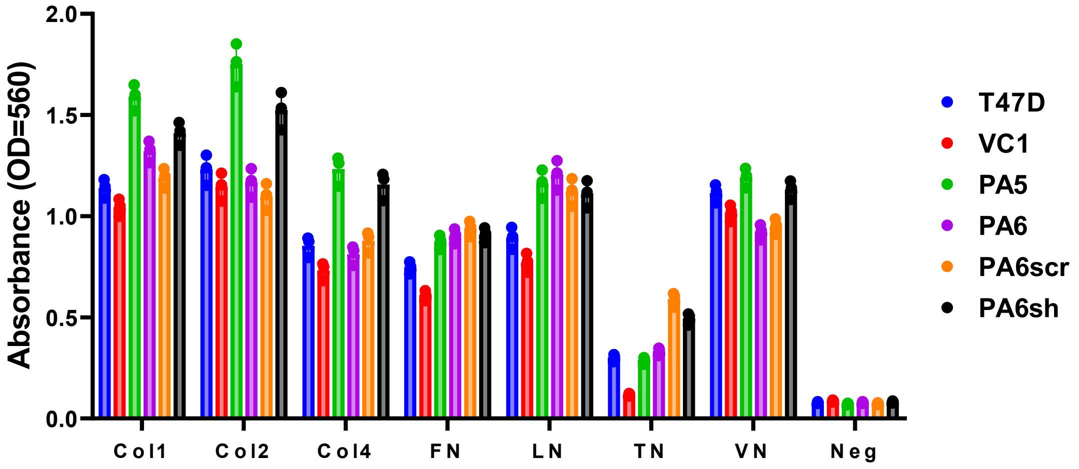

Col1, collagen I
Col2, collagen II
Col4, collagen IV
FN, fibronectin
LN, laminin
TN, tenascin
VN, vitronectin
Neg, negative control (BSA)

Figure S16 Effect of PTH-AS expression on ECM adhesion of T47D cells. ECM adhesion assay. Cells are seeded on ECM-coated 96-well plates and cultured for 48 h. The absorbance of lysate of the stained adherent cells is measured at 570 nm and normalized to the BSA value of each cell (*n* = 3). All data are shown as the mean ± SD.

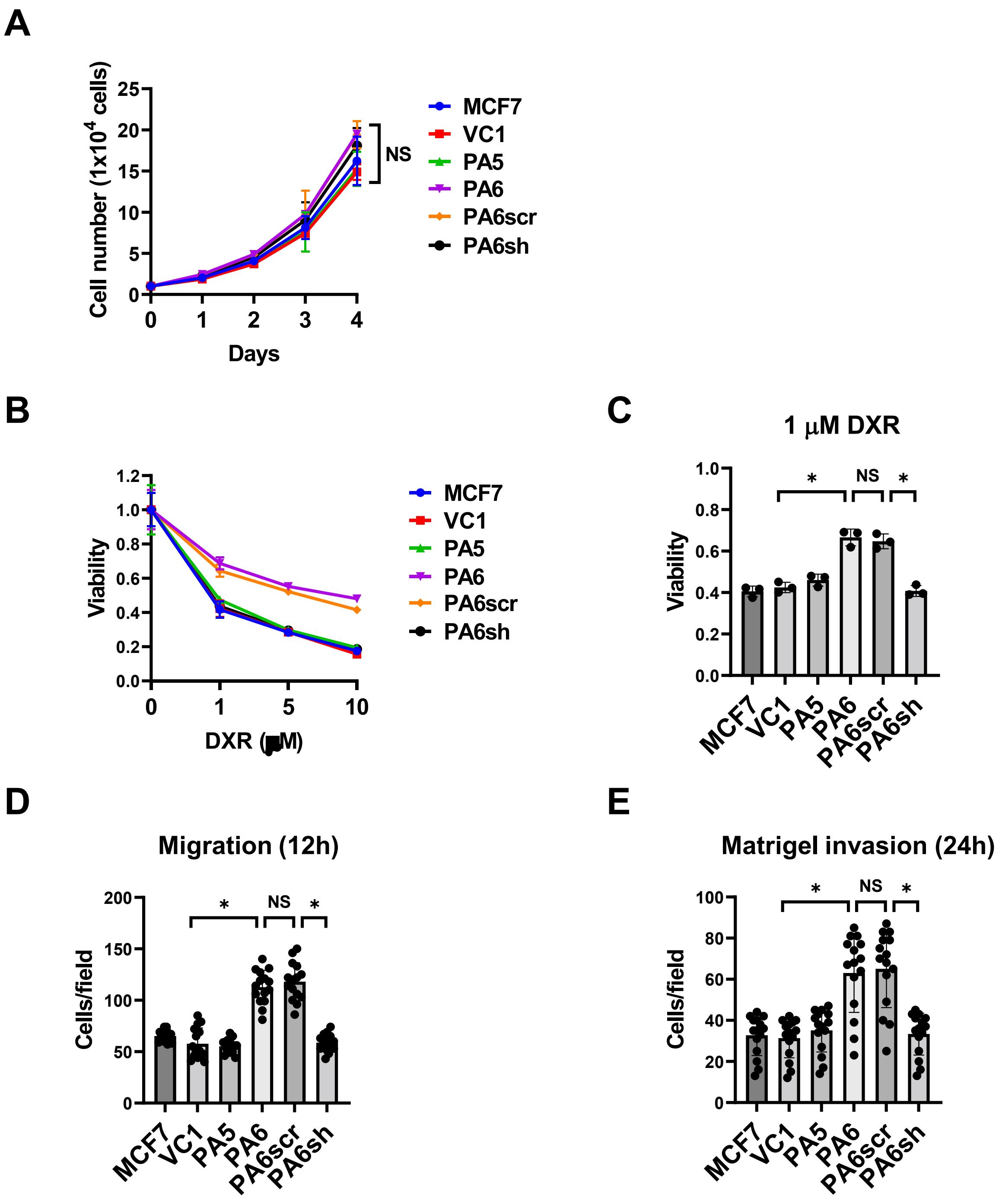

Figure S17 Effects of PTH-AS expression on malignant properties of MCF7 cells. *A*, Cell proliferation. Cells (1×10^4) were seeded, and the number of viable cells was counted after 2-, 4- and 6-days using trypan blue exclusion (n = 3). *B* and *C*, Resistance to DXR. Cells (5×10^5) were treated with DXR at the indicated concentrations (*B*) or 1 μM (*C*) for 48 h, and cell viability is assessed by MTT assay. The viability of DXR-untreated cells is shown as 1. *D*, migration assay. *E*, Matrigel invasion assay. After culturing for 12 hours or 24 hours, the infiltrating cells were stained and counted by the same method as in Fig. 4*I*-*L* (n = 15). All data are shown as the mean ± SD. NS, not significant. *P < 0.05 by one-way ANOVA.

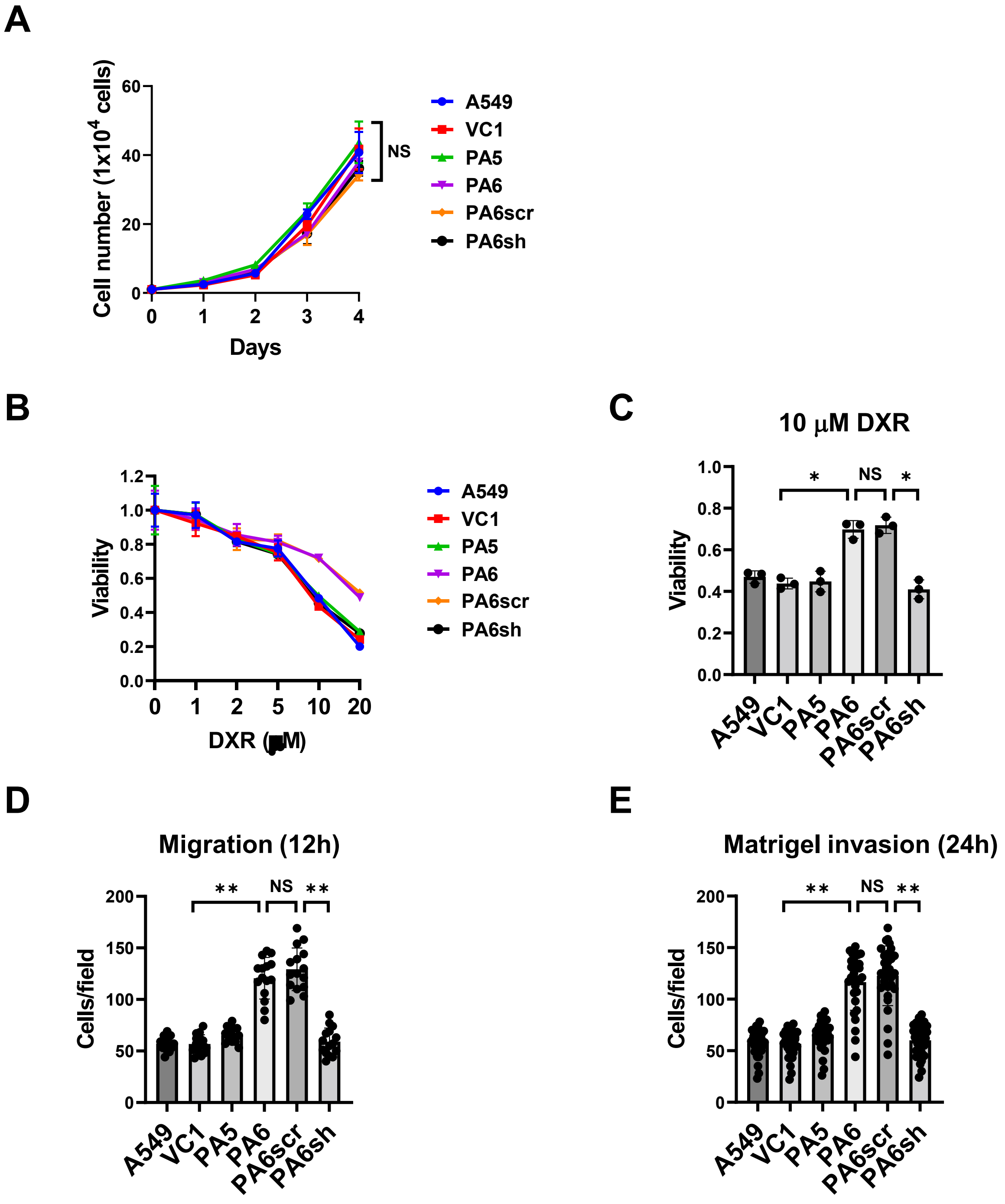

Figure S18 Effects of PTH-AS expression on malignant properties of A549 cells. *A*, Cell proliferation. Cells (1 × 10^4) were seeded, and the number of viable cells was counted after 2-, 4- and 6-days using trypan blue exclusion (n = 3). *B* and *C*, Resistance to DXR. Cells (5 × 10^5) were treated with DXR at the indicated concentrations (*B*) or 10 µM (*C*) for 48 h, and cell viability is assessed by MTT assay. The viability of DXR-untreated cells is shown as 1. *D*, migration assay. *E*, Matrigel invasion assay. After culturing for 12 hours or 24 hours, the infiltrating cells were stained and counted by the same method as in Fig. 4*I-L* (n = 15). All data are shown as the mean ± SD. NS, not significant. *P < 0.05, **P < 0.01 by one-way ANOVA.

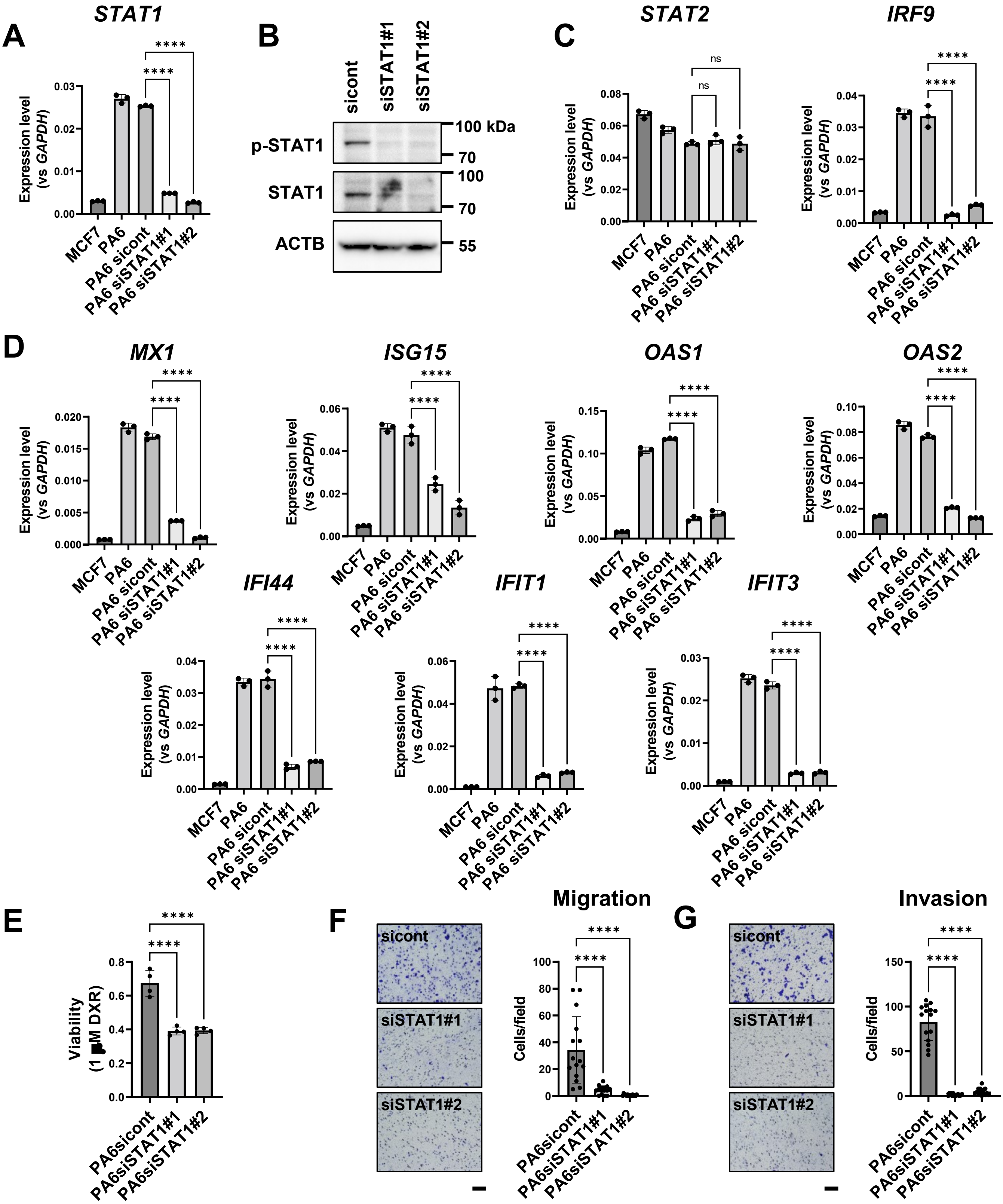

Figure S19 Effects of STAT1 knockdown on gene expression and cancer characteristics in MCF7-PA6 cells. *A* and *B*, Confirmation of siRNA-mediated STAT1 knockdown in MCF7-PA6 cells by qRT-PCR (n = 3) (*A*) and Western blotting analysis (*B*). The uncropped images are shown in Supporting information S26. *C* and *D*, Analysis of gene expression levels associated with STAT1 knockdown in MCF7-PA6 cells by qRT-PCR (n = 3). mRNA levels of JAK-STAT signaling related genes (*C*) and IRDS genes (*D*). *E*, Resistance to DXR. Each cell (5×10^5) was treated with 1 μM DXR for 48 hours and cell viability was assessed by MTT assay. The DXR-untreated survival rate of each cell was calculated as 1 (n = 4). *F*, Transwell Migration Assay. *G*, Matrigel Invasion Assay. After culturing for 12 hours for the migration assay and 24 hours for the invasion assay, the infiltrating cells were stained with crystal violet. The stained cells were observed under a bright field (100×), and the number of cells per field was counted (n = 15). Scale bars, 100 μm. Data are shown as the mean ± SD. ns, not significant. ****P < 0.0001 by one-way ANOVA.

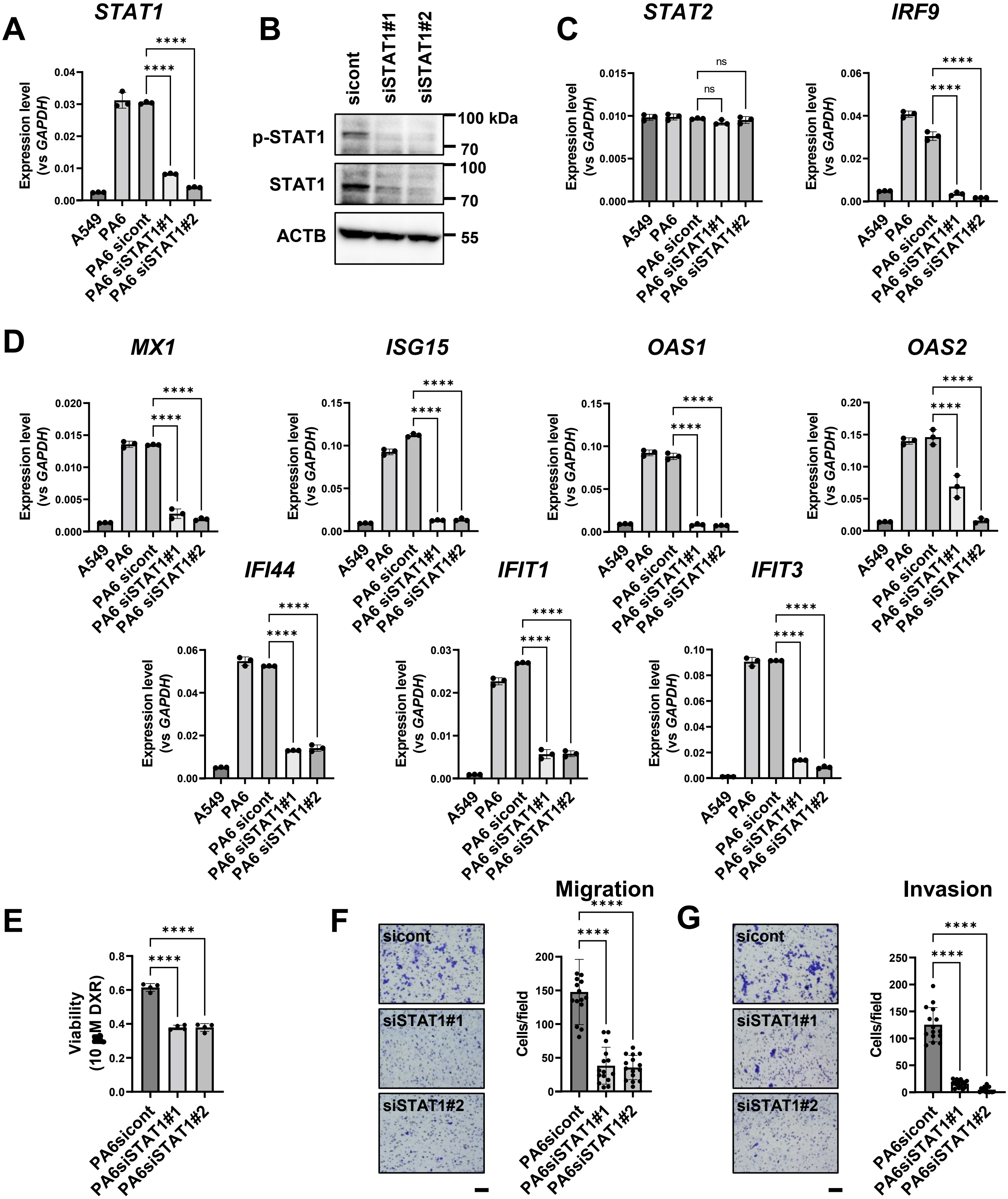

Figure S20 Effects of STAT1 knockdown on gene expression and cancer characteristics in A549-PA6 cells. *A* and *B*, Confirmation of siRNA-mediated STAT1 knockdown in MCF7-PA6 cells by qRT-PCR (*n* = 3) (*A*) and Western blotting analysis (*B*). The uncropped images are shown in Supporting information S26. *C* and *D*, Analysis of gene expression levels associated with STAT1 knockdown in A549-PA6 cells by qRT-PCR (*n* = 3). mRNA levels of JAK-STAT signaling related genes (*C*) and IRDS genes (*D*). *E*, Resistance to DXR. Each cell (5×10^5) was treated with 10 μM DXR for 48 hours and cell viability was assessed by MTT assay. The DXR-untreated survival rate of each cell was calculated as 1 (*n* = 4). *F*, Transwell Migration Assay. *G*, Matrigel Invasion Assay. After culturing for 12 hours for the migration assay and 24 hours for the invasion assay, the infiltrating cells were stained with crystal violet. The stained cells were observed under a bright field (100×), and the number of cells per field was counted (*n* = 15). Scale bars, 100 μm. Data are shown as the mean ± SD. ns, not significant. ****$P < 0.0001$ by one-way ANOVA.

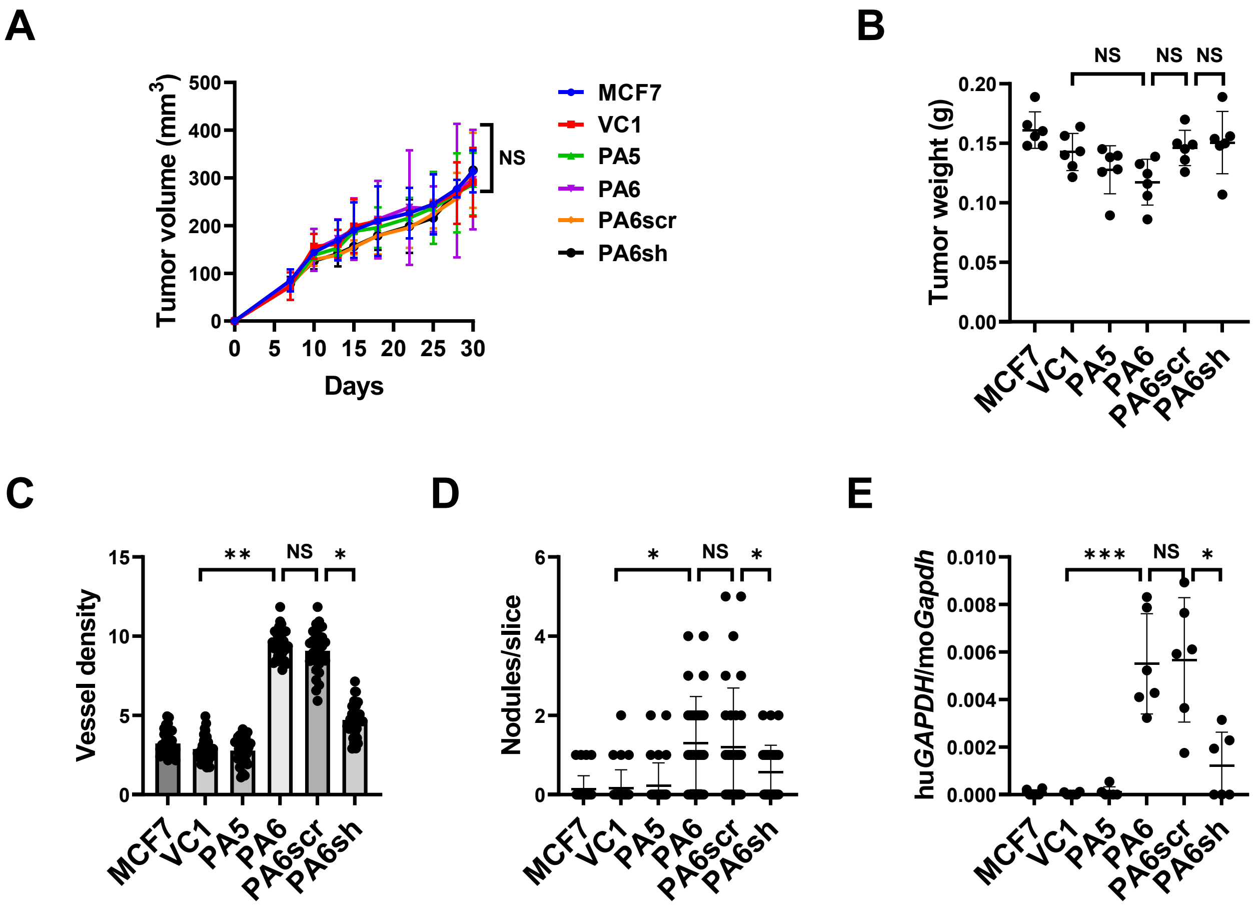

F

	MCF7	VC1	PA5	PA6	PA6scr	PA6sh
**Tumor**	6/6 (100 %)	6/6 (100 %)	6/6 (100 %)	6/6 (100 %)	6/6 (100 %)	6/6 (100 %)
**Lung meta**	2/6 (33.3 %)	2/6 (33.3 %)	2/6 (33.3 %)	6/6 (100 %)	6/6 (100 %)	3/6 (33.3 %)

Figure S21 Effects of PTH-AS expression on MCF7 tumor growth and lung metastasis. The effect of PTH-AS expression on MCF7 cells on tumor growth and lung metastasis was quantified and evaluated by the same method as in Fig.5. *A*, Tumor growth (n = 6). *B*, Tumor weight (n = 6). *C,* Tumor blood vessel density (n = 30). *D*. Metastatic nodules per lung (n = 30). *E*, Evaluation of lung metastasis by qRT-PCR (n = 6). All data are shown as the mean ± SD. **P* < 0.05, ***P* < 0.01, ****P* < 0.001 by one-way ANOVA.

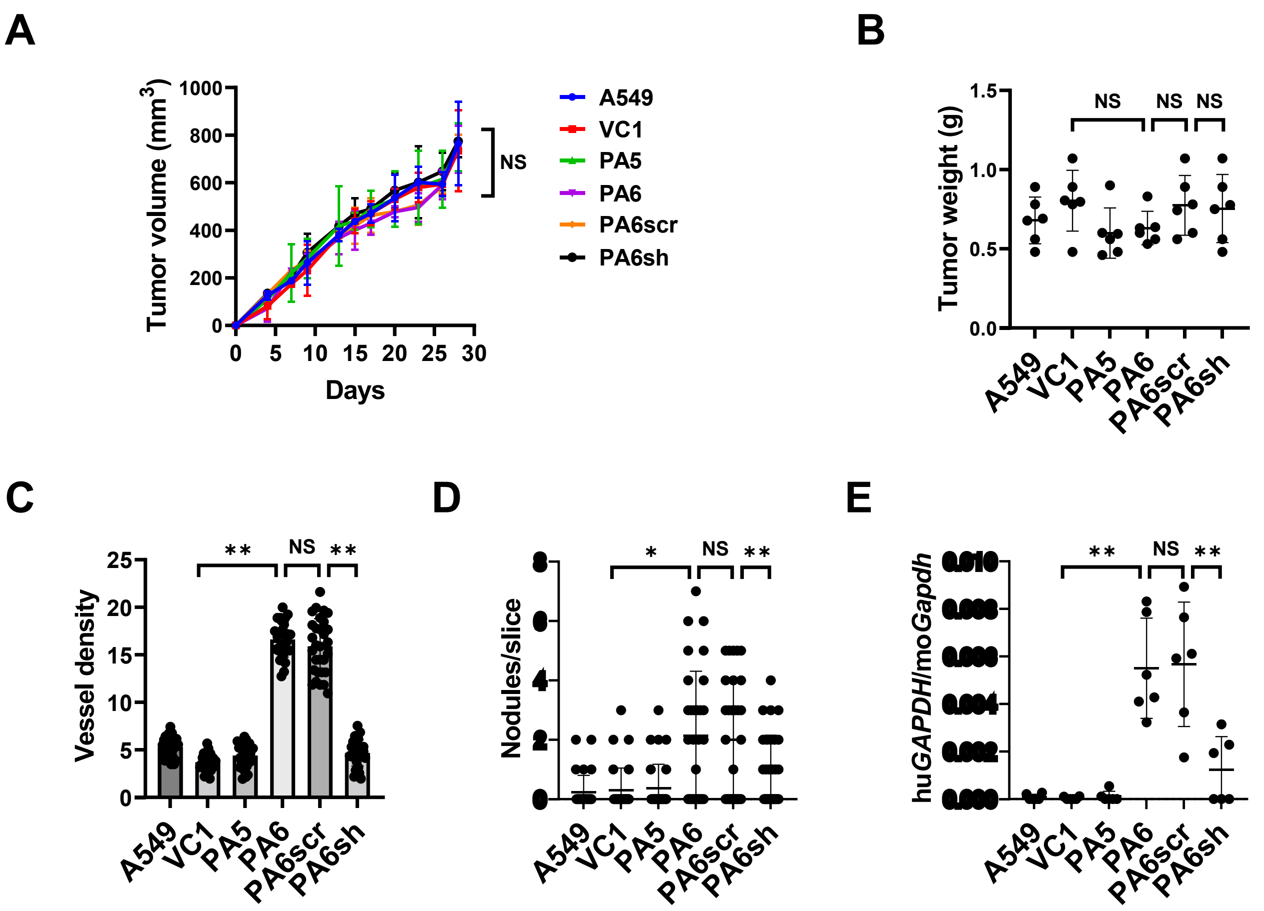

F

	A549	VC1	PA5	PA6	PA6scr	PA6sh
**Tumor**	6/6 (100 %)	6/6 (100 %)	6/6 (100 %)	6/6 (100 %)	6/6 (100 %)	6/6 (100 %)
**Lung meta**	2/6 (33.3 %)	2/6 (33.3 %)	2/6 (33.3 %)	6/6 (100 %)	6/6 (100 %)	3/6 (33.3 %)

Figure S22 Effects of PTH-AS expression on A549 tumor growth and lung metastasis. The effect of PTH-AS expression on A549 cells on tumor growth and lung metastasis was quantified and evaluated by the same method as in Fig.5. *A*, Tumor growth (n = 6). *B*, Tumor weight (n = 6). *C,* Tumor blood vessel density (n = 30). *D*. Metastatic nodules per lung (n = 30). *E*, Evaluation of lung metastasis by qRT-PCR (n = 6). All data are shown as the mean ± SD. *P < 0.05, **P < 0.01 by one-way ANOVA.

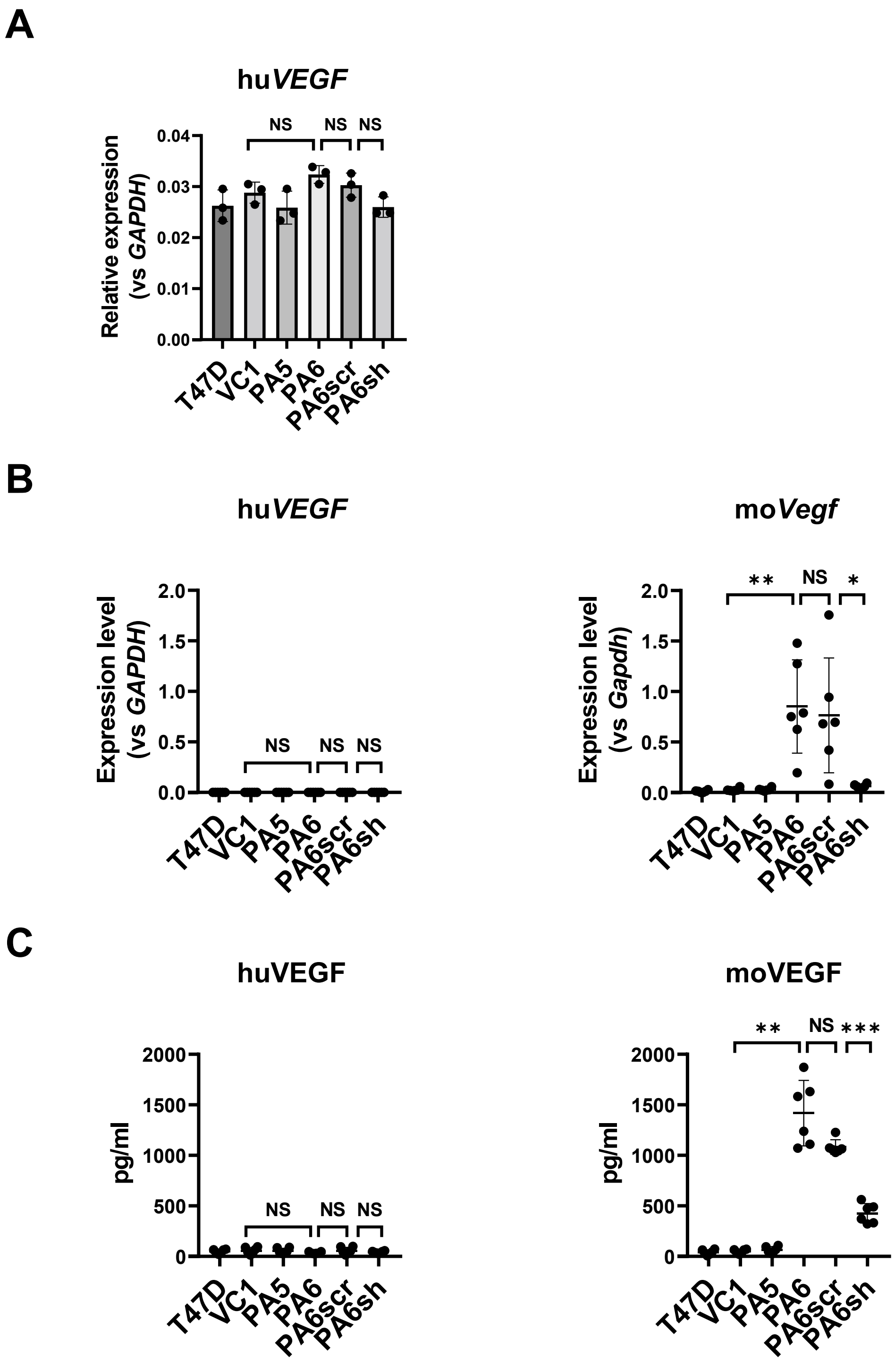

Figure S23 ***VEGF*** **levels in T47D cells or T47D xenografts expressing PTH-AS.** *A*. VEGF mRNA levels in T47D-VC1, T47D-PA5, and T47D-PA6 cells (*n* = 3). *B*. Human and mouse mRNA levels in xenografts (*n* = 6). For normalization, GADPH primers common to humans and mice were used. *C*. Human or mouse VEGF protein concentration in tumor tissue. Tumor protein extracts were prepared and human or mouse VEGF protein concentrations were measured by ELISA. Each data was normalized to total protein concentration. All data are shown as the mean ± SD. NS, not significant. **P* < 0.05, ***P* < 0.01 , ****P* < 0.001 by one-way ANOVA.

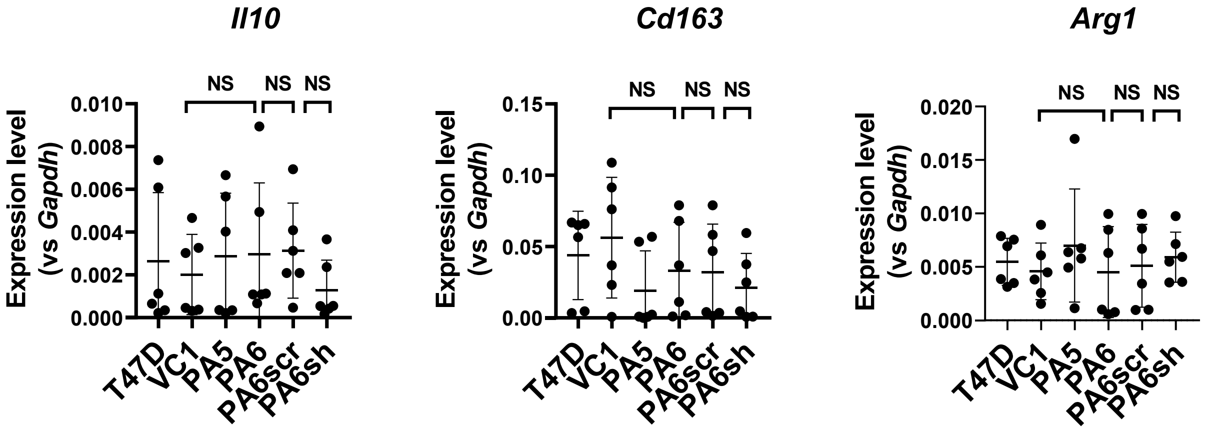

Figure S24 Expression levels of M2 markers in xenografts. The expression level of each gene were examined by qRT-PCR using RNA extracted from the tumor (n = 6). For normalization, *GAPDH* primers common to human and mice were used. The expression levels of the M1 markers are shown in Fig. 7J. Data are shown as the mean ± SD. NS, not significant by one-way ANOVA.

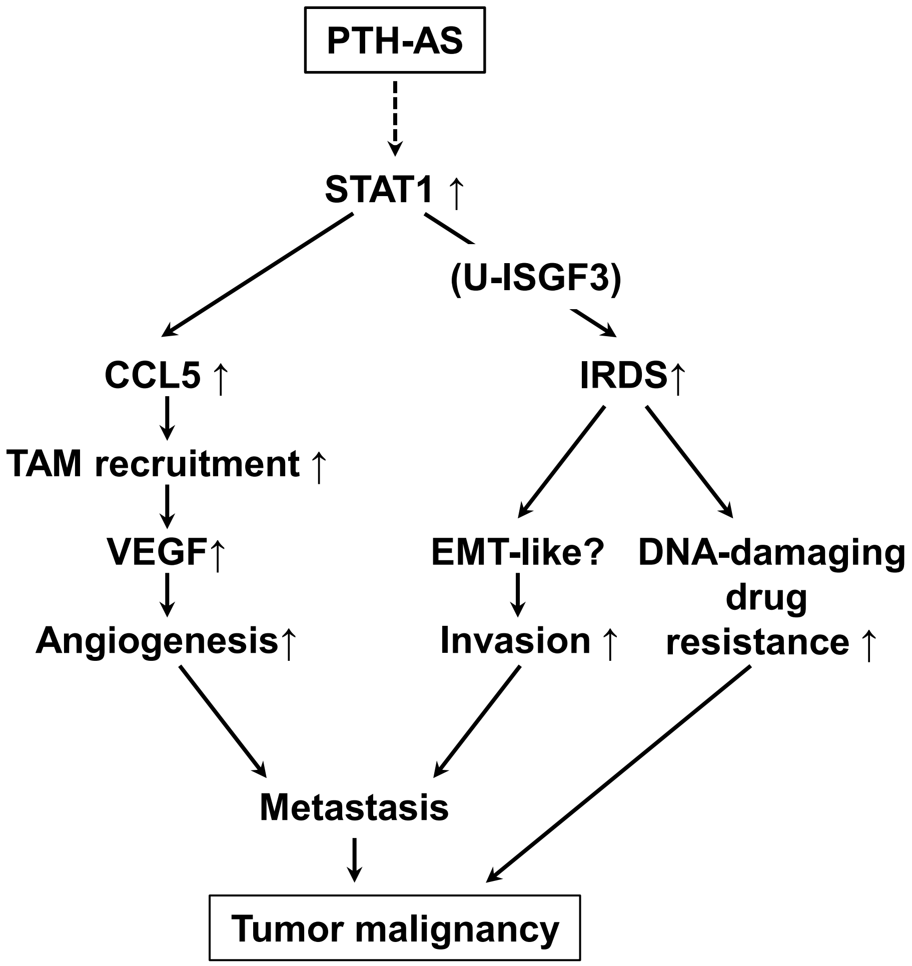

Figure S25 Summary of possible effects of PTH-AS expression on tumor malignancy.

Fig. 3D

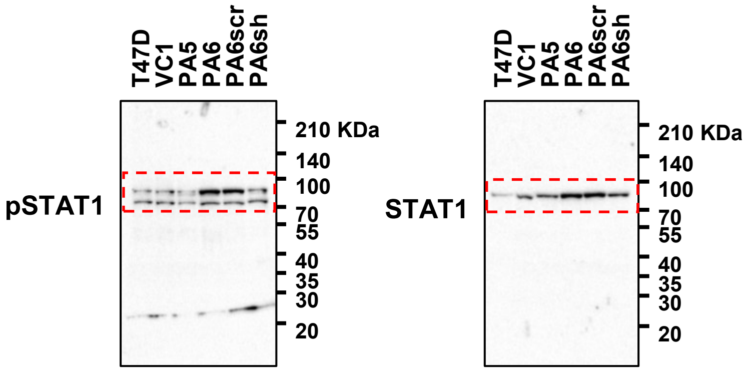

Figs. 5*B*, S19*B* and S20*B*

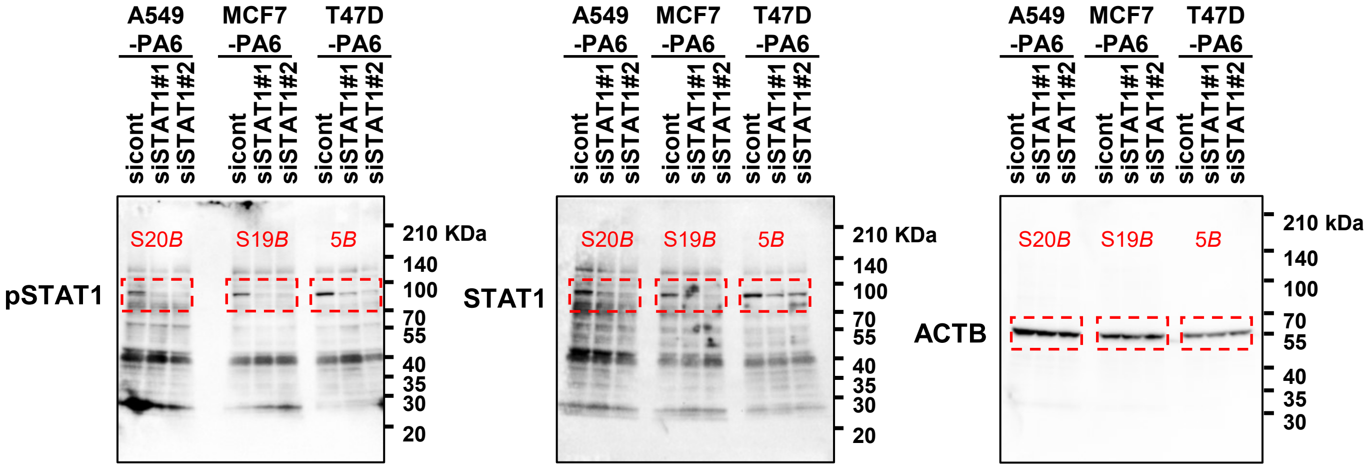

Fig. 7*C*

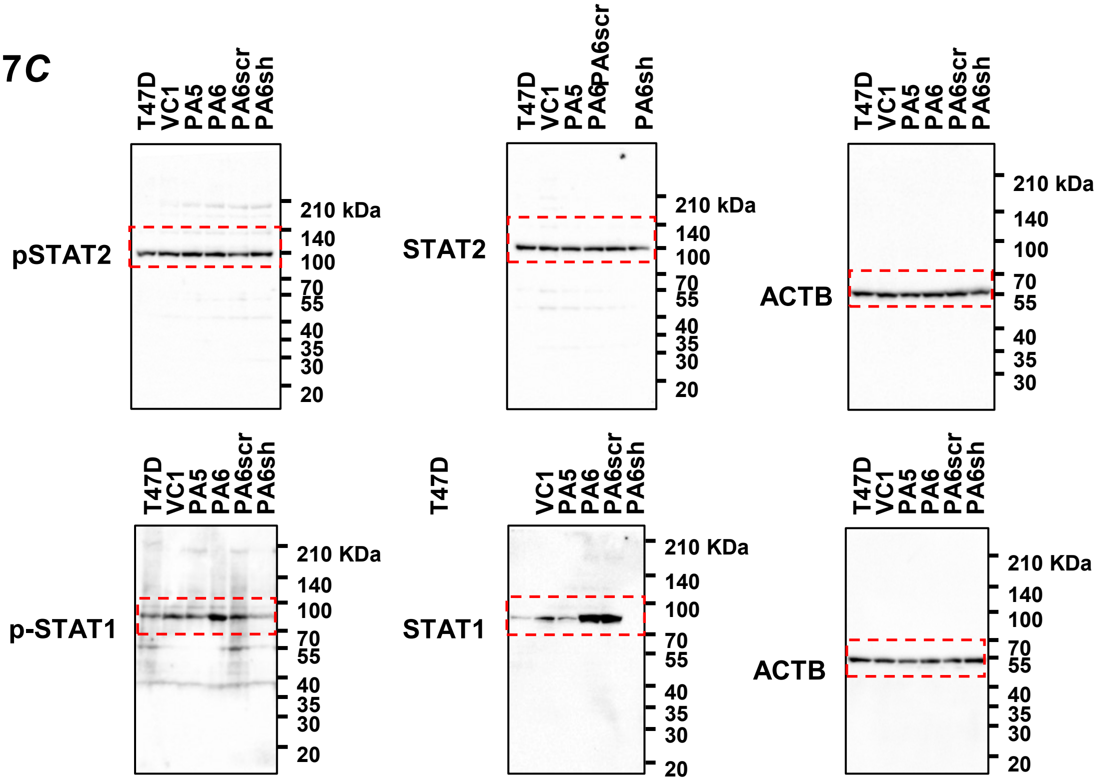

Figure S26 Full version images of western blotting. The cropped images (red dotted line) are shown in Figs. 3*D,* 5*B*, S19*B*, S20*B* and 7*C*.
